# Nutations in Plant Shoots: Endogenous and Exogenous Factors in the Presence of Mechanical Deformations

**DOI:** 10.3389/fpls.2021.608005

**Published:** 2021-03-23

**Authors:** Daniele Agostinelli, Antonio DeSimone, Giovanni Noselli

**Affiliations:** ^1^SISSA–International School for Advanced Studies, Trieste, Italy; ^2^The BioRobotics Institute, Scuola Superiore Sant'Anna, Pisa, Italy

**Keywords:** plant morphogenesis, 3D morphoelastic rods, differential growth, circumnutation, two-oscillator hypothesis

## Abstract

We present a three-dimensional morphoelastic rod model capable to describe the morphogenesis of growing plant shoots driven by differential growth. We discuss the evolution laws for endogenous oscillators, straightening mechanisms, and reorientations to directional cues, such as gravitropic reactions governed by the avalanche dynamics of statoliths. We use this model to investigate the role of elastic deflections due to gravity loading in circumnutating plant shoots. We show that, in the absence of endogenous cues, pendular and circular oscillations arise as a critical length is attained, thus suggesting the occurrence of an instability triggered by exogenous factors. When also oscillations due to endogenous cues are present, their weight relative to those associated with the instability varies in time as the shoot length and other biomechanical properties change. Thanks to the simultaneous occurrence of these two oscillatory mechanisms, we are able to reproduce a variety of complex behaviors, including trochoid-like patterns, which evolve into circular orbits as the shoot length increases, and the amplitude of the exogenous oscillations becomes dominant.

## 1. Introduction

**Figure 1:**
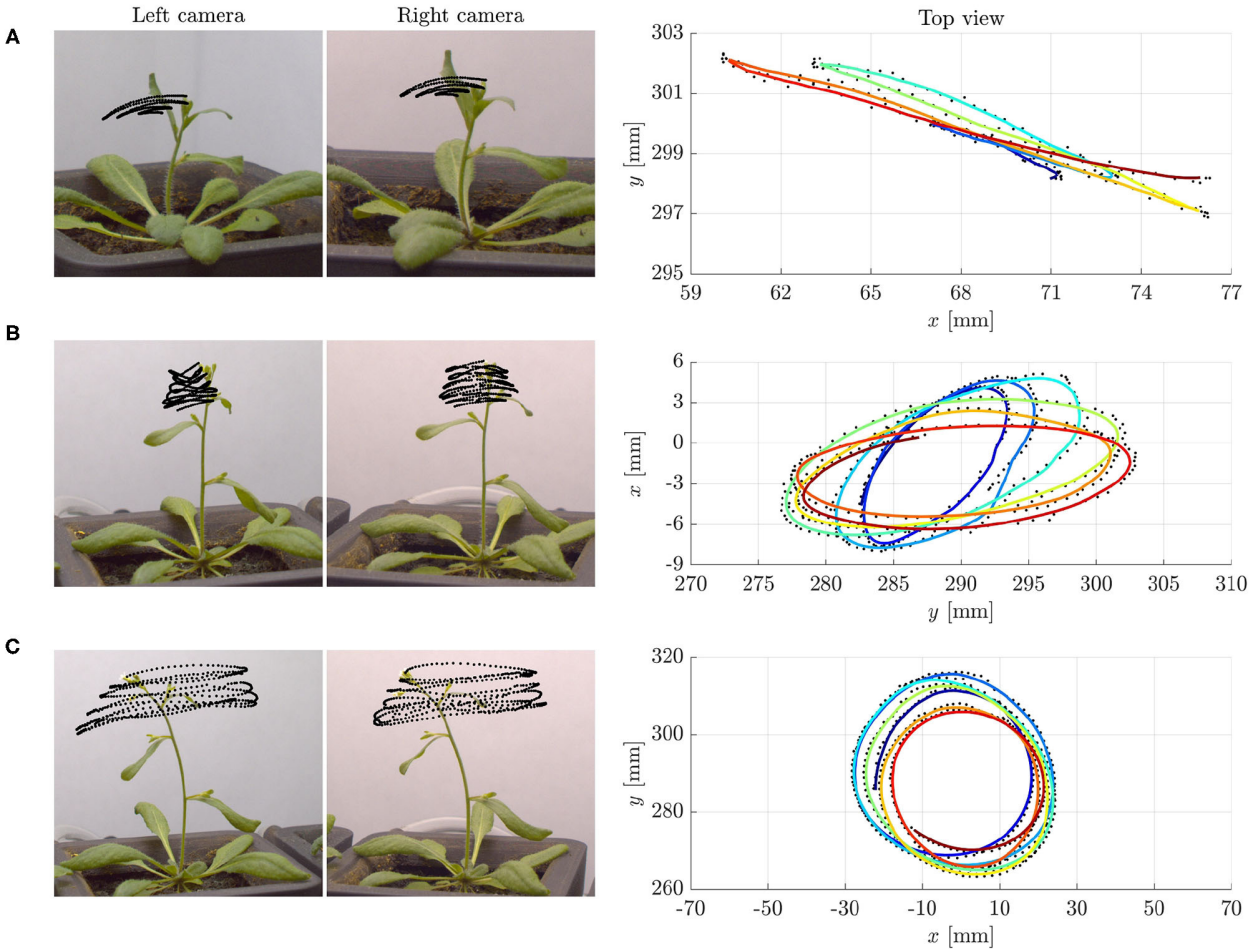
Examples of tip trajectories from specimens of *Arabidopsis thaliana* (ecotype Col-0) grown under normal gravity conditions (1 g) and continuous light at the SAMBA laboratory of SISSA: **(A)** Pendular oscillations in specimen 1 (about 27 days old), **(B)** elliptic, and **(C)** circular patterns in specimen 2 (about 29 days old). Left: Stereo pair of images corresponding to the last instant of the tip trajectories. The superimposed black dots are the tracked positions of the tip at time intervals of 1 min. Right: Top view of the tip trajectories as reconstructed by matching corresponding points in the stereo pair of images. The colored lines, from blue to red for increasing time, are obtained by moving averaging over ten detected positions, shown in black. Notice that the characteristic time of circumnutational oscillations τ_*c*_ is of the order of 70–90 min. We refer to Supplementary Material (section S5 of Data Sheet 1 and Data Sheet 2) for more details on the experiments.

The extraordinary variety of movements in plants has fascinated scientists since the pioneering work by Darwin (1880), and is raising considerable and growing interest. Many essential functions involve passive conformational changes and active adaptation triggered by diverse conditions. Spectacular illustrations range from reproductive methods by explosive seed and pollen dispersal (Hofhuis et al., 2016), to nutrition and defense strategies, such as the snapping of *Venus flytrap* (Forterre et al., 2005) or the closing of *Mimosa Pudica*. This also includes the search for mechanical support by circumnutations of aerial organs in climbing plants, namely, pendular, elliptical or circular oscillatory movements. However, nutations occur also in some non-climbing plants, such as *Arabidopsis thaliana*, without any obvious purpose (see Figure 1 and Video 1).

The nature of plant circumnutations has been intensively investigated over the last century, and this produced three main hypotheses (Stolarz, 2009). First, as already suggested by Darwin (1880), endogenous oscillators might drive the observed oscillatory movements, by internally regulating differential growth. Second, circumnutations might be the byproduct of posture control mechanisms that overshoot the target equilibrium, due to delayed responses (Gradmann, 1922). Third, the previous two mechanisms might be combined in a “two-oscillator” hypothesis in which endogenous prescriptions and delayed responses coexist (Johnsson et al., 1999; Stolarz, 2009).

The overshooting hypothesis is typically based on externally driven feedback systems (of gravitropic, autotropic, phototropic, or other nature) and neglects mechanical (elastic) deformations of the plant organ. In this way, the role of elasticity in plant circumnutations remained relatively unexplored until a recent study showed that accounting for elastic deflections due to gravity loading enriches the scenario. Spontaneous oscillations might arise as system instabilities (bifurcations) when a loading parameter exceeds a critical value (Agostinelli et al., 2020b), similarly to dynamic instabilities (*flutter*) exhibited, e.g., by mechanical systems under nonconservative loads (Bigoni and Noselli, 2011; Bigoni et al., 2018). However, such results are restricted to the two-dimensional case, and derive from a simplified analysis at constant length and without proprioceptive responses.

Plant circumnutations have often been studied by tracking the trajectory of the apical part of a growing shoot. It has also been recognized, however, that in order to relate these measurements to the inner mechanism of plants, the relation between the shape of the whole organ and the position of the apical tip needs to be resolved (Bastien and Meroz, 2016). It is then quite natural to ask the question whether light on such a relation can be shed by a model of the shoot as a growing rod, capable of deforming elastically under the action of external loads, and responding actively to external stimuli. To test this hypothesis, and building upon the theory of morphoelastic rods (Goriely, 2017), we propose in the present study a three-dimensional model for growing plant shoots that includes gravitropic responses driven by the statoliths avalanche dynamics, proprioception, lignification, and also an endogenous oscillator. Our goal is to apply the model to the study of plant circumnutations, with a particular focus on the issue that has received least attention until now, namely, the role of elastic deformations in determining the observed movements.

We find that, in our model, elastic deformations significantly impact the growth regimes for which spontaneous (exogenous) oscillations arise in the absence of endogenous factors. Indeed, when all the biomechanical and growth parameters but the stiffness and the shoot length are fixed, there exists a critical length beyond which spontaneous oscillatory motions, representing a system instability due to the presence of gravity loading, become possible. Moreover, we show that when an endogenous factor is also present, the relative weight of endogenous versus exogenous mechanisms changes: as the shoot elongates, the oscillations associated with the instability mechanism become dominant over those of endogenous origin (see Videos 2, 3). In intermediate regimes, we find trochoid-like patterns that are reminiscent of the trajectories observed by Schuster and Engelmann (1997) in the hypocotyls of *A. thaliana*. In this way, the combination of endogenous and exogenous factors in the presence of mechanical (elastic) deformations might reproduce the observed variability of nutation patterns as a consequence of the existence of different regimes, and of their interplay. Therefore, the present study suggests that it may be worth re-examining the “two-oscillator hypothesis” from a renewed perspective, namely, by accounting for elastic deformations due to gravity loading.

## 2. Materials and Methods

In this section we propose a 3D morphoelastic rod model to describe a growing plant shoot and we derive a reduced model for times that are short compared to those characterizing growth. Here we provide a minimal description of the model and we refer to Supplementary Material (section S1) for a detailed derivation of the proposed equations from a more general framework.

### 2.1. The Morphoelastic Rod Model for Growing Plant Shoots

**Figure 2:**
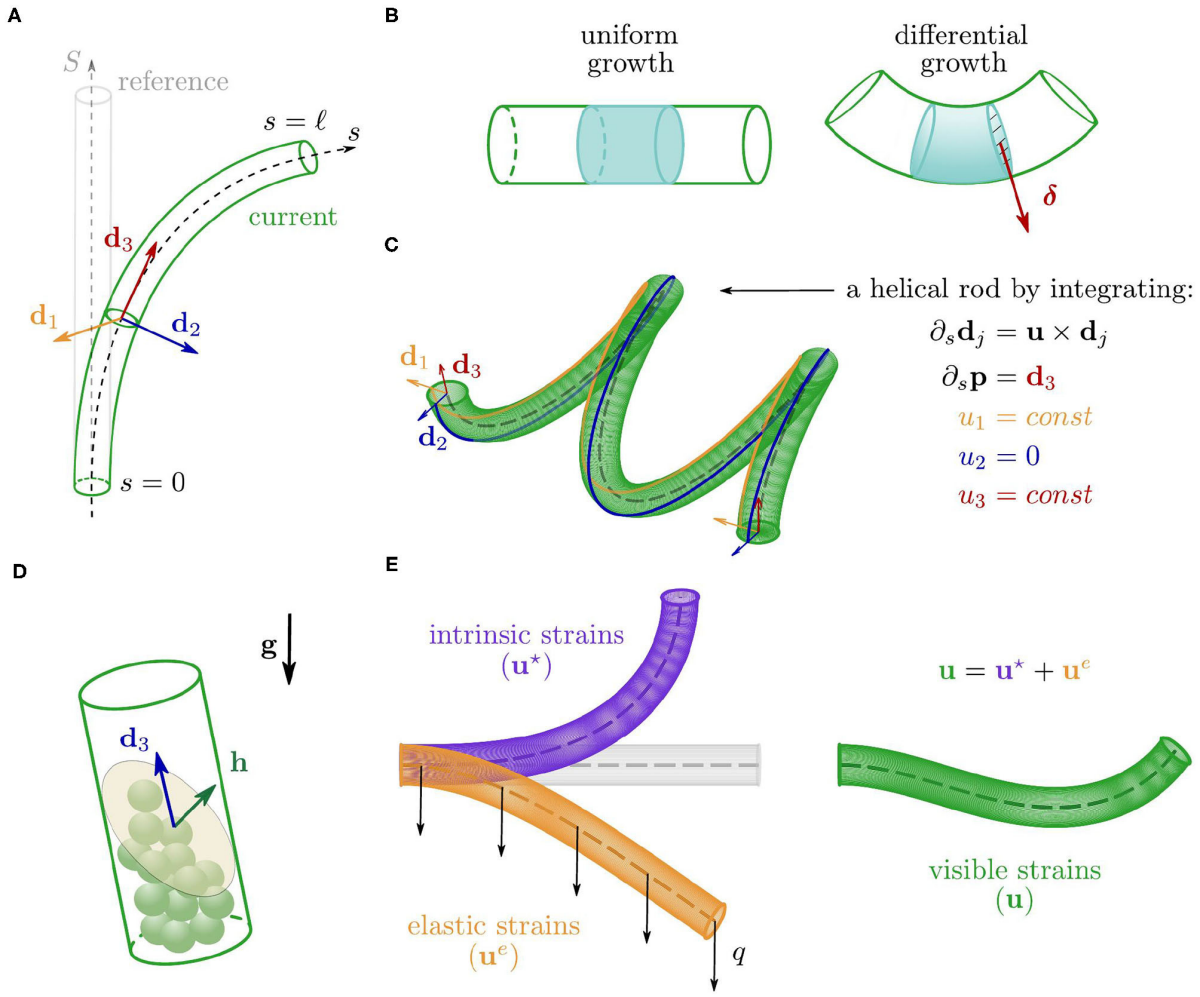
**(A)** Configurations of the cylindrical rod, with the centerline (dashed) and the orthonormal basis of directors associated with a cross section. **(B)** Comparison between two portions of the rod undergoing a uniform growth (left) and a linear differential growth with gradient **δ** (right). **(C)** Example of helical configuration obtained by integrating Equations (1) for a constant strain vector **u**. **(D)** Sketch of a single statocyte cell where **h** is the average outer normal to the free surface of the pile of statoliths. **(E)** Additive decomposition of the visible strains in elastic and intrinsic contributions.

We model a plant shoot as a growing, unshearable, and (elastically) inextensible elastic rod with circular cross section of radius *r*. This is a slender three-dimensional solid body that at time *t* occupies the cylindrical region surrounding a *centerline* **p**(*s, t*) ∈ ℝ^3^ where *s* ∈ [0, ℓ(*t*)] is the arc length parameter and identifies material cross sections, from the base, *s* = 0, to the apex, *s* = ℓ. In order to describe the motion of the rod, we equip the centerline with an orthonormal basis of *directors* {**d**_1_(*s, t*), **d**_2_(*s, t*), **d**_3_(*s, t*)}, which define the orientation of each cross section. In particular, **d**_1_ and **d**_2_ identify two material fibers of the cross section, and **d**_3_: = **d**_1_ × **d**_2_ is the unit vector normal to the cross section and tangent to the centerline, i.e., **d**_3_ = ∂_*s*_**p** where ∂_*s*_ denotes the partial derivative with respect to *s* (see Figure 2A, Antman, 2005). The *Darboux vector* **u**(*s, t*) describes the way the directors **d**_*i*_ vary along the rod through ∂_*s*_**d**_*j*_ = **u** × **d**_*j*_ for *j* = 1, 2, 3, and it encodes the bending and torsional deformations of the rod (see Figure 2C). The components *u*_*j*_: = **u**·**d**_*j*_, *j* = 1, 2, are called *flexural strains* as they define the bending of the rod about **d**_1_ and **d**_2_, whereas *u*_3_ = **u**·**d**_3_ is the *torsional strain*, as it defines the relative rotation about **d**_3_ between neighboring cross sections (Antman, 2005). At any time *t*, we reconstruct the rod—centerline and directors—corresponding to a Darboux vector **u** by integrating the kinematic equations

(1) $$\begin{array}{l} \partial_{s}{\text{d}}_{j}=\text{u}\times {\text{d}}_{j}\text{ for}j=1,2,3,\text{ and }{\text{d}}_{3}=\partial_{s}\text{p}, \end{array}$$

see Figure 2C.

The deformations encoded in **u** result from both an elastic component due to external loads, and a spontaneous or intrinsic one (**u**^⋆^, see Figure 2E) associated with growth. In the absence of external loads, the Darboux vector results only from the intrinsic component (namely, **u** = **u**^⋆^) and the spontaneous strains completely define the configuration of the rod through (1). We discuss first the intrinsic term, and then the elastic one.

**Figure 3:**
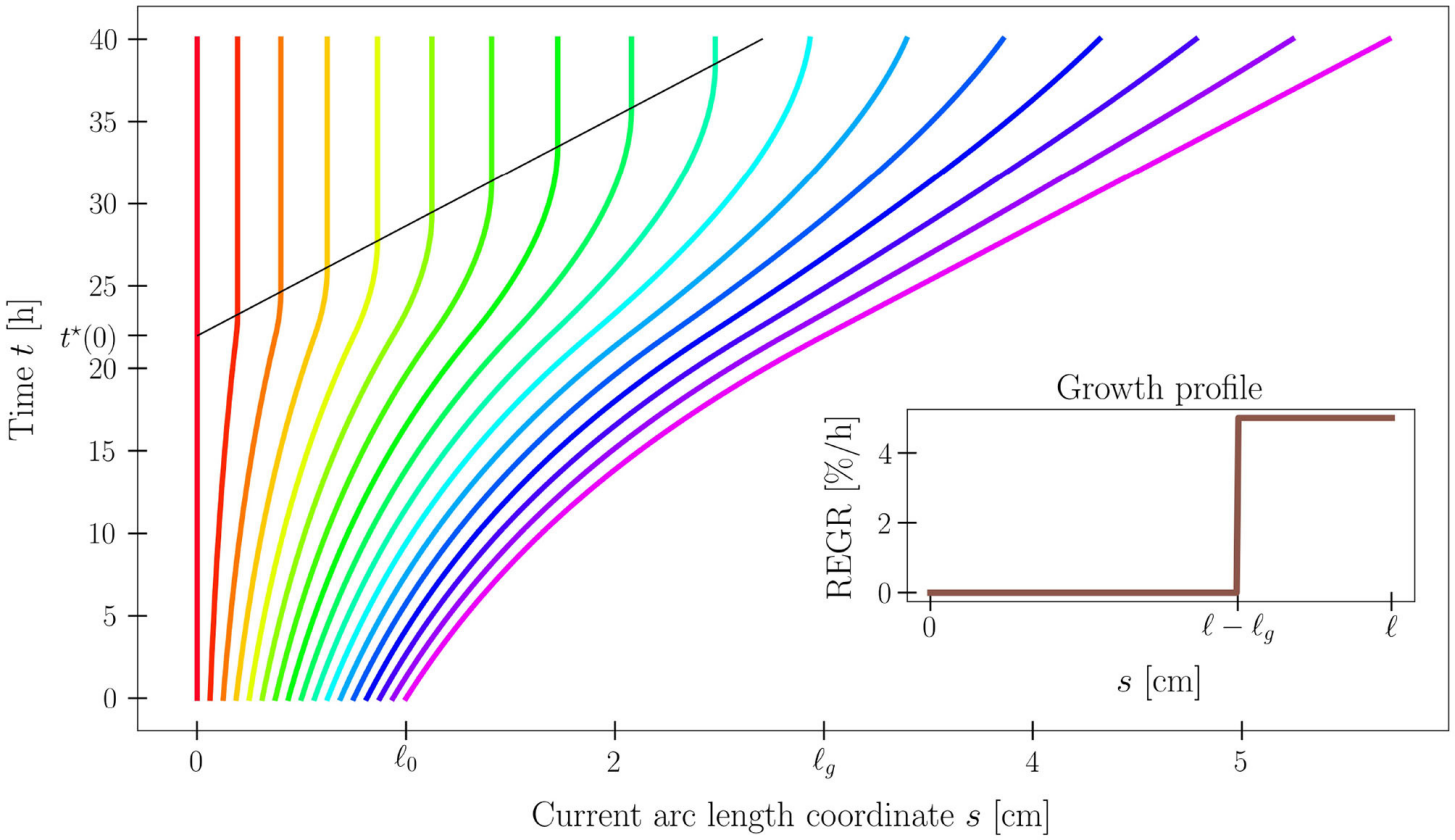
Time evolution of the current arc length *s*(*S, t*) solving (2)–(3), for a set of 17 material points along the plant organ. Model parameters are ℓ_0_ = 1 cm, ℓ_*g*_ = 3 cm, and τ_*g*_ = 20 h. Notice the black line that denotes the time t^*^(*S*) at which the material point *S* exits the growth zone, as its distance from the tip exceeds ℓ_*g*_. The analytical expression of *s* is reported in Supplementary Material (section S1.3).

We describe the subapical growth of the shoot as a stretch of the centerline with respect to a reference configuration that is parameterized by an arc length coordinate *S* ∈ [0, ℓ_0_], where ℓ_0_ is the plant length at initial time. We define the *stretch* as γ: = ∂_*S*_*s* where *s*(*S, t*) is the coordinate at time *t* of the material cross section identified by the parameter *S* in the reference configuration. Then we define the *true strain* as ε: = ln γ, so that the *relative elemental growth rate* (REGR) introduced by Erickson and Sax (1956) reduces to $\overset{∙}{\varepsilon }=\partial_{t}\gamma /\gamma$, where a superimposed dot denotes the material time derivative. Since such a quantity can be experimentally measured by tracking material markers along the organ (Maksymowych et al., 1985; Berg and Peacock, 1992; Mullen et al., 1998; van der Weele et al., 2003; Hall and Ellis, 2012; Phyo et al., 2017), we prescribe a function REGR(*s, t*), vanishing outside the elongating zone of length ℓ_*g*_, i.e., [ℓ(*t*) − ℓ_*g*_, ℓ(*t*)], and such that $\overset{∙}{\varepsilon }=\mathrm{REGR}$. Consequently, subapical growth is governed by two coupled PDEs, namely,

(2) $$\begin{array}{l} \frac{1}{\gamma }\frac{\partial \gamma }{\partial t}=\mathrm{REGR}\text{ and }\gamma =\frac{\partial s}{\partial S}, \end{array}$$

to be solved for *S* ∈ [0, ℓ_0_] and *t* ≥ 0, with initial condition γ (·, 0)≡1 and fixed boundary datum *s*(0, ·)≡0. Following previous studies (Bastien et al., 2014; Chelakkot and Mahadevan, 2017), among the possible choices (Agostinelli et al., in press), we use a piecewise constant growth function, namely,

(3) $$\mathrm{REGR}(S,t)=\{\begin{array}{ll} 0 & \text{if }s(S,t)\leq \ell (t)-\ell_{g},\\ \frac{1}{\tau_{g}} & \text{if }s(S,t)>\ell (t)-\ell_{g}, \end{array}$$

where τ_*g*_ > 0 is the characteristic growth time. Problem (2)–(3) has an analytical solution *s*(*S, t*), and the shoot length ℓ(*t*) grows linearly in time, attaining the growth length ℓ_*g*_ (see Figure 3).

While growing, the shoot evolves and adapts its shape by means of a spatially nonhomogeneous growth rate of the cross section. This morphing mechanism, referred to as *differential growth*, allows the plant to accommodate to a variety of stimuli. We assume that the organ radius *r* is small enough to justify a linearization about the center of the cross section, leading to a growth rate that has a linear profile with gradient **δ**, which relates to the spontaneous strains as follows

(4) $$\begin{array}{l} \delta \left(s,t\right)={\overset{∙}{u}}_{1}^{⋆}\left(s,t\right){\text{d}}_{2}\left(s,t\right)-{\overset{∙}{u}}_{2}^{⋆}\left(s,t\right){\text{d}}_{1}\left(s,t\right), \end{array}$$

where $u_{j}^{⋆}$ are the components of **u**^⋆^ with respect to the local basis {**d**_*j*_}, i.e., $u_{j}^{⋆}:={\text{u}}^{⋆}\cdot {\text{d}}_{j}$, for *j* = 1, 2, 3. Equation (4) reveals that differential growth governs the evolution of the spontaneous strains, $u_{j}^{⋆}$. Indeed, the growth gradient **δ** defines the direction along which the strain rate (or REGR) is linearly distributed on the cross section (see Figure 2B), thus determining the rates of the flexural strains, ${\overset{∙}{u}}_{1}^{⋆}$ and ${\overset{∙}{u}}_{2}^{⋆}$. Here we assume that $u_{3}^{⋆}\equiv 0$, even though this torsional strain could play a crucial role in other growth mechanisms, such as those observed in twining plants. In this study, we consider three possible drivers for differential growth: an endogenous oscillator, gravitropic responses, and proprioception. In the following, we discuss the evolution laws of the spontaneous strains as individually produced by each of these mechanisms.

1. *Endogenous oscillator*. Several studies on plant growth and nutations have revealed a strong correlation between oscillatory movements and biological rhythms, thus supporting the Darwinian concept of *internal oscillator* (Berg and Peacock, 1992; Schuster and Engelmann, 1997; Shabala and Newman, 1997; Buda et al., 2003; Shabala, 2003; Niinuma et al., 2005; Mugnai et al., 2015). Here, following previous approaches (Bastien and Meroz, 2016; Porat et al., 2020), we implement a spatially uniform time-harmonic oscillator with period τ_*e*_, namely,
  (5a) $$\begin{array}{l} {\overset{∙}{u}}_{1}^{⋆}\left(s,t\right)=\alpha \frac{\overset{∙}{\varepsilon }\left(s,t\right)}{r}\cos\left(2\pi t/\tau_{e}\right), \end{array}$$
  (5b) $$\begin{array}{l} {\overset{∙}{u}}_{2}^{⋆}\left(s,t\right)=\alpha \frac{\overset{∙}{\varepsilon }\left(s,t\right)}{r}\sin\left(2\pi t/\tau_{e}\right), \end{array}$$
  where α is a nonnegative dimensionless sensitivity associated with the endogenous cues.
2. *Gravitropic reactions*. Plant organs sense gravity through the sedimentation of starch-filled plastids, called *statoliths*, into specialized cells, called *statocytes*, which are found along the shoot growing zone and in the root caps (Chauvet et al., 2019; Nakamura et al., 2019). By extending the approach taken by Chauvet et al. (2019) to the three-dimensional case, we model the statoliths free surface as a plane with normal **h**, whose dynamics is a viscous relaxation to −**g** (see Figure 2D). As shown in Supplementary Material (section S2), the time evolution of **h** is governed by
  (6) $$\begin{array}{l} \underset{j}{\sum }{ḣ}_{j}{\text{d}}_{j}=\frac{1}{\tau_{a}}\text{h}\times \left(\text{h}\times \text{g}\right), \end{array}$$
  where τ_*a*_ is the characteristic time for the statoliths avalanche dynamics and *h*_*j*_: = **h**·**d**_*j*_. Equation (6) shows that **h** = −**g** provides an equilibrium configuration for the statoliths. Then we implement a gravitropic contribution to the growth gradient to align the axis of the organ with the perceived gravity vector **h** as
  (7a) $$\begin{array}{l} {\overset{∙}{u}}_{1}^{⋆}\left(s,t\right)=-\beta \frac{\overset{∙}{\varepsilon }\left(s,t\right)}{r\tau_{m}}\int_{-\infty }^{t-\tau_{r}}e^{-\frac{1}{\tau_{m}}\left(t-\tau_{r}-\tau \right)}h_{2}\left(s,\tau \right)\text{d}\tau , \end{array}$$
  (7b) $$\begin{array}{l} {\overset{∙}{u}}_{2}^{⋆}\left(s,t\right)=\beta \frac{\overset{∙}{\varepsilon }\left(s,t\right)}{r\tau_{m}}\int_{-\infty }^{t-\tau_{r}}e^{-\frac{1}{\tau_{m}}\left(t-\tau_{r}-\tau \right)}h_{1}\left(s,\tau \right)\text{d}\tau , \end{array}$$
  where β is a nonnegative dimensionless constant expressing the organ sensitivity to gravi-stimulation, whereas τ_*m*_ and τ_*r*_ are the memory and reaction times of the gravitropic response, respectively. Equations (7) account for memory and delay effects by means of an integration over time of the stimulus, properly weighted by an exponential function (Israelsson and Johnsson, 1967; Chauvet et al., 2019; Agostinelli et al., 2020b). If we confine the rod to a plane, Equations (7) reduce to the extension of Sach's sine law provided by Chauvet et al. (2019).
3. *Straightening mechanisms* (or *proprioception*). Some experiments have pointed out the existence of an independent straightening mechanism, often referred to as proprioception, autotropism, or autostraightening, which is triggered by bending of the organ (Okamoto et al., 2015). Following Bastien et al. (2014) and Bastien and Meroz (2016), we assume that such a straightening response is driven by the geometric curvature of the organ, i.e., $\kappa ={\left(u_{1}^{2}+u_{2}^{2}\right)}^{1/2}$, thus producing a growth gradient parallel to the visible normal vector $\nu :=\kappa^{-1}\partial_{s}{\text{d}}_{3}$. This leads to the evolution laws
  (8a) $$\begin{array}{l} {\overset{∙}{u}}_{1}^{⋆}\left(s,t\right)=-\eta \frac{\overset{∙}{\varepsilon }\left(s,t\right)}{{\bar{\tau }}_{m}}\int_{-\infty }^{t-{\bar{\tau }}_{r}}e^{-\frac{1}{{\bar{\tau }}_{m}}\left(t-{\bar{\tau }}_{r}-\tau \right)}u_{1}\left(s,\tau \right)\text{d}\tau , \end{array}$$
  (8b) $$\begin{array}{l} {\overset{∙}{u}}_{2}^{⋆}\left(s,t\right)=-\eta \frac{\overset{∙}{\varepsilon }\left(s,t\right)}{{\bar{\tau }}_{m}}\int_{-\infty }^{t-{\bar{\tau }}_{r}}e^{-\frac{1}{{\bar{\tau }}_{m}}\left(t-{\bar{\tau }}_{r}-\tau \right)}u_{2}\left(s,\tau \right)\text{d}\tau , \end{array}$$
  where η is a nonnegative dimensionless constant for the proprioceptive sensitivity of the organ, whereas ${\bar{\tau }}_{m}$ and ${\bar{\tau }}_{r}$ are the memory and reaction times of the straightening mechanism, respectively. As for gravitropism, we model proprioception by a distributed delay with an exponential kernel.

We assume the existence of separate signaling pathways for different simultaneous stimuli so that we obtain the overall evolution laws for the spontaneous strains by summing the corresponding right-hand sides of Equations (5), (7), and (8). In fact, studies show interactions between different tropisms (Correll and Kiss, 2002), but we neglect them because the microscopic mechanisms and pathways for this cross-talk remain unknown for many plant responses (Okamoto et al., 2015; Su et al., 2017; Levernier et al., 2021).

For both gravitropism and proprioception, we provide a phenomenological description of memory and delay by integrating the stimuli over time, weighted by an exponential kernel function [cf. Equations (7)–(8)]. Even though we could implement more realistic models by fitting the kernel function with suitable experiments (Meroz et al., 2019), or by solving for the hormone transport (Moulton et al., 2020; Levernier et al., 2021), we opt for parsimony over complexity in order to capture the phenomenon while making it mathematically tractable.

Finally, we adapt this framework to include elastic deformations due to gravity loading. In this case, the configuration of the rod results from two contributions: elastic and intrinsic strains (see Figure 2E). The additive formula captures the simplest form consistent with this idea, so that

(9) $$\begin{array}{l} u_{j}=u_{j}^{⋆}+u_{j}^{e}\text{ for}j=1,2,3, \end{array}$$

where $u_{j}^{⋆}$ are the spontaneous strains that evolve to accommodate to external cues, as previously discussed, whereas $u_{j}^{e}$ are the elastic strains due to bending and twisting moments generated by the gravity loading. In particular, we assume

(10) $$\begin{array}{l} u_{j}^{e}=\frac{m_{j}}{K_{j}}\text{ for}j=1,2,3, \end{array}$$

where *K*_*j*_ and *m*_*j*_: = **m**·**d**_*j*_ are the bending and torsional stiffnesses and moments, respectively, and **m** is the contact couple. Under the assumption that the time scale for mechanical equilibrium is much shorter than any biological time scale of the plant, we determine the contact couple **m** by solving the two fundamental equations of mechanical balance, i.e.,

(11) $$\begin{array}{l} \partial_{s}\text{n}\left(s,t\right)+\text{f}\left(s,t\right)=0,\;\partial_{s}\text{m}\left(s,t\right)+\partial_{s}\text{p}\left(s,t\right)\times \text{n}\left(s,t\right)=0, \end{array}$$

where **n** is the contact force acting on the cross section, and **f** is the body force per unit current length. Equations (11) derive from the balance laws of linear and angular momentum under the assumption of negligible inertia effects, and they are referred to as the *Kirchhoff equations* (Antman, 2005; Goriely, 2017). More specifically, we assume that the shoot carries a uniform distributed gravity load *q* = ρ*gA* ≥ 0, where ρ is the mass density, *A* = π*r*^2^ is the cross-sectional area, and *g* is the gravitational acceleration so that **f** = *q***g**. Since the apical end is free, the boundary conditions associated with Equations (11) read **n**(ℓ(*t*), *t*) = **0** and **m**(ℓ(*t*), *t*) = **0**. Then, the first equation can be integrated to get **n** = *q*(ℓ(*t*) − *s*)**g** and we are left with

(12) $$\begin{array}{l} \partial_{s}\text{m}\left(s,t\right)=q\left(\ell \left(t\right)-s\right)\text{g}\times {\text{d}}_{3}\left(s,t\right), \end{array}$$

together with the boundary condition **m**(ℓ(*t*), *t*) = **0**. In principle, once **m** is known, using the constitutive Equations (10), one could express the strains as **u** = **u**^⋆^ + **u**^*e*^ and obtain, by integration of the kinematic Equations (1), the configuration of the rod. However, Equation (12) cannot be solved independently, since it contains the unknown tangent **d**_3_ and determining the visible configuration of the rod requires, in fact, the solution of a coupled nonlinear system. In the case **h** = −**g** of fast statoliths avalanche dynamics, these are 24 scalar equations [i.e., (1), (9), (10), (12), and the three equations determining the evolution of $u_{j}^{⋆}$] in 24 scalar variables (the components of **p**, **d**_1_, **d**_2_, **d**_3_, **u**, **u**^⋆^, **u**^*e*^, and **m**). Neglecting elasticity, this reduces to 15 equations in 15 scalar variables (the components of **p**, **d**_1_, **d**_2_, **d**_3_, and **u** = **u**^⋆^).

The stiffnesses *K*_*j*_ depend on the cross section geometry. Even though elliptic cross sections might provide a better approximation (Paul-Victor and Rowe, 2011), we opt for the simpler assumption of circular cross section of radius *r*. In this case, *K*_1_ = *K*_2_ = *EI* where *E* is the Young's modulus and *I* = π*r*^4^/4 is the second moment of inertia, and *K*_3_ = μ*J* where *J* = 2*I* and μ = 2*E*(1 + ν) is the shear modulus determined by the Poisson's ratio ν (Antman, 2005; Goriely, 2017). Since we expect lignification processes to play a crucial role in the mechanics of the organ, over a long period of time, we include also a rod stiffening, by adapting the approach of Chelakkot and Mahadevan (2017); in particular, we assume the Young's modulus to evolve in time according to

(13) $$\begin{array}{l} E\left(S,t\right)=E_{1}-\left(E_{1}-E_{0}\right)e^{-\max\left\{0,t-t^{⋆}\left(S\right)\right\}/\tau_{\ell }}, \end{array}$$

where *t*^⋆^(*S*) is the time at which the material point *S* exits the growth zone, τ_ℓ_ is the lignification characteristic time, whereas *E*_0_ and *E*_1_ are the minimum and maximum values of the Young's modulus, respectively.

### 2.2. The Reduced Model for Short Times

The rod model introduced in section 2.1 presents some difficulties for a theoretical study but we can derive a reduced version for a linearized analysis of processes that are much faster than growth (and hence lignification) and much slower than the statoliths dynamics. Indeed—for short time periods—we can neglect changes in length (i.e., ℓ ≈ ℓ_0_ ≤ ℓ_*g*_), and in the Young's modulus (i.e., *E* ≈ *E*_0_), as growth and lignification are slow; in this case, current and reference arc lengths coincide such that material time derivatives reduce to standard time derivatives. Moreover, we can disregard the transient in the statoliths sedimentation and assume that, at all times, the statoliths free surface is a plane normal to the gravity vector (i.e., **h** = −**g**), as its dynamics is fast. We report the equations of such a model in Supplementary Material (section S3.3), together with their linearization for the stability analysis.

## 3. Results

In this section we present the results of our study of plant circumnutations based on the models proposed in section 2, and for the relevant parameters listed in Table 1. The reduced model reveals the possible emergence of spontaneous oscillations associated with gravitropic and proprioceptive responses as a mechanism independent of endogenous oscillators; the onset of these spontaneous oscillations is crucially affected by elasticity. The full model shows the effects of shoot elongation during growth, and confirms the potential key role of elasticity in circumnutations: a loading parameter, such as the shoot length, determines the relative importance between the two oscillatory mechanisms.

**Table 1:** Summary of model parameters and respective order of magnitude of their values.

**Parameter**	**Description**	**Value**	**References**
**Sensitivities for differential growth**			
α	Sensitivity to endogenous cues	0−1	Assumed
β	Gravitropic sensitivity	0.8	Chauvet et al. (2019)
η	Proprioceptive sensitivity	20	Assumed
**Characteristic times**			
τ_*a*_	Time for statoliths avalanche	2 min	Chauvet et al. (2019)
τ_*e*_	Period of endogenous oscillations	20 min	Assumed
τ_*m*_	Gravitropic memory time	12 min	Chauvet et al. (2019)
${\bar{\tau }}_{m}$	Proprioceptive memory time	12 min	Assumed
τ_*r*_	Gravitropic reaction time	12 min	Chauvet et al. (2019)
${\bar{\tau }}_{r}$	Proprioceptive reaction time	12 min	Assumed
τ_*g*_	Growth time	20−40 h	Chauvet et al. (2019), Hall and Ellis (2012), and Phyo et al. (2017)
τ_ℓ_	Lignification time	6 d	Assumed
**Morphological and biomechanical parameters**			
*r*	Radius of the cross section	0.5 mm	Paul-Victor and Rowe (2011)
ℓ_*g*_	Growth zone	4−7 cm	Hall and Ellis (2012)
ν	Poisson's ratio	0.5	Assumed
ρ	Mass density	10^3^ Kg m^−3^	Chelakkot and Mahadevan (2017)
*E*_0_	Initial Young's modulus	10 MPa	Chelakkot and Mahadevan (2017)
*E*_1_/*E*_0_	Stiffening ratio due to lignification	200	Assumed

### 3.1. The Regime of Short Times

As detailed in Supplementary Material (section S3), a linearized analysis of the reduced model reveals the change in the stability character of the upright trivial equilibrium when a loading parameter exceeds a critical value. In the nonlinear model, this event corresponds to the emergence of periodic, oscillatory movements, as found numerically by means of the computational model described in Supplementary Material (section S4). We investigate the role of elasticity in determining the stability thresholds for the onset of spontaneous oscillations in the following different scenarios.

**Figure 4:**
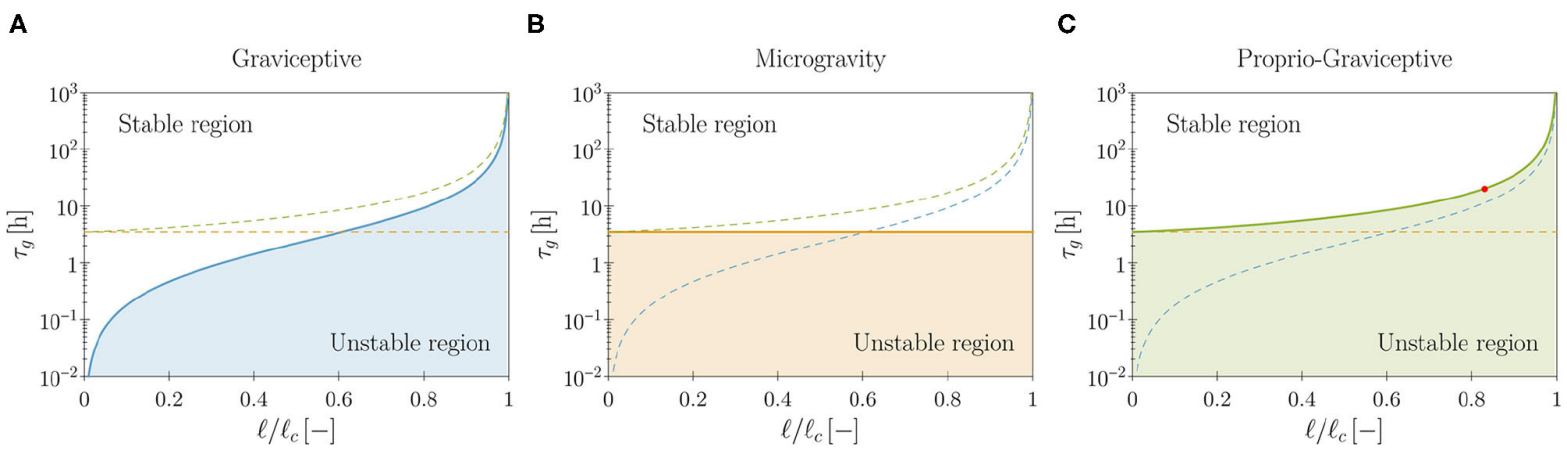
Theoretical stability boundaries in terms of the model parameters (τ_*g*_, ℓ). Blue, orange and green curves are for the graviceptive case (α = η = 0, β = 0.8), for microgravity (α = β = 0, η = 20, *q* = 0) and for the proprio-graviceptive case (α = 0, β = 0.8, η = 20), respectively. In each plot **(A–C)** results for the relevant case are reported as solid curves, whereas the boundaries for the other two cases are shown as dashed curves for comparison purposes. Model parameters are those reported in Table 1. Shoot length ℓ is normalized by the self-buckling length $\ell_{c}:=\sqrt[{3}]{\alpha_{0}EI/q}$ where α_0_ ≈ 7.837 (Greenhill, 1881). The red dot in **(C)** corresponds to the computational results of Figure 5.

**Figure 5:**
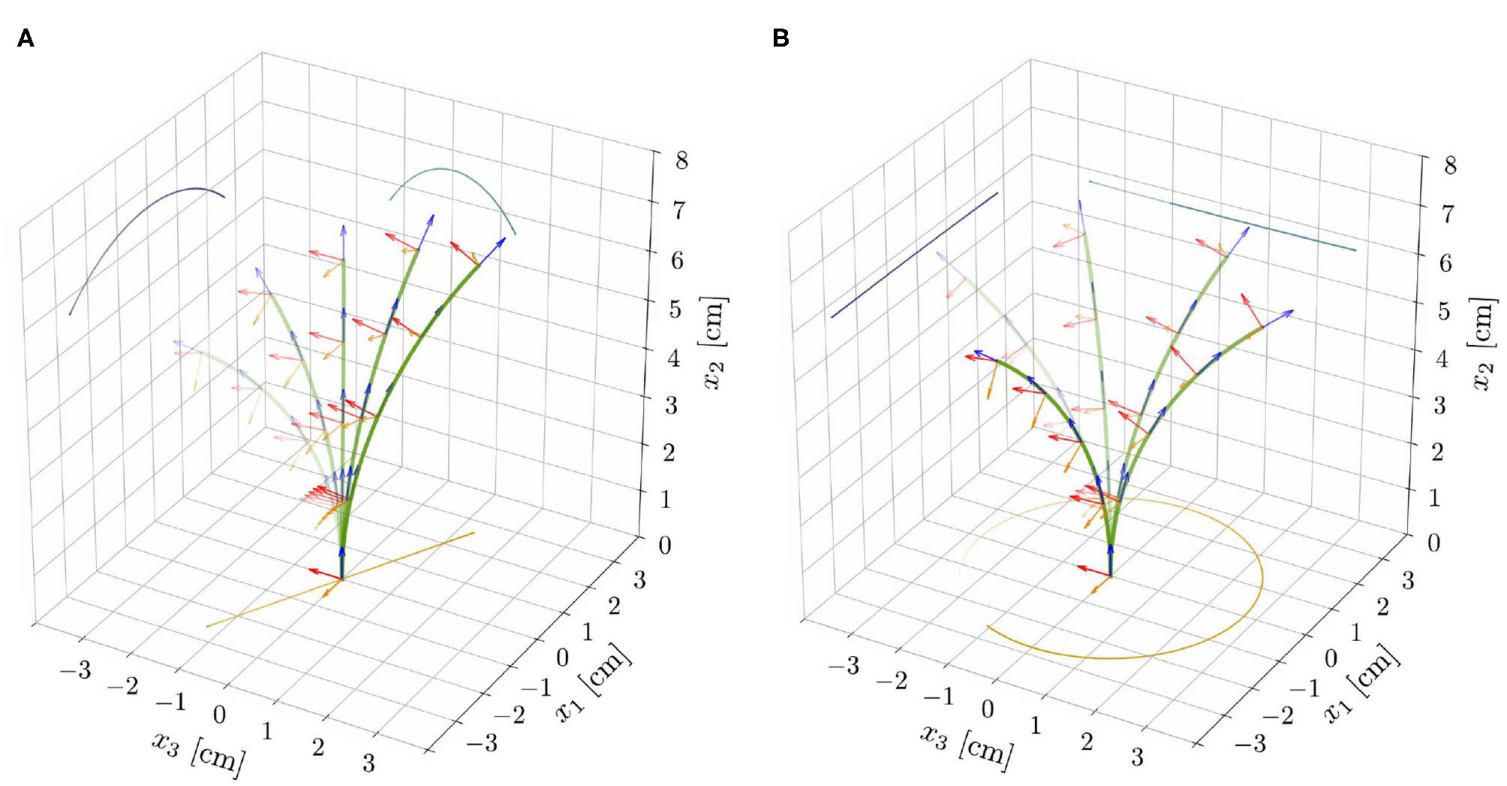
Superposition of deformed shapes and respective directors, from the reduced nonlinear rod model for ℓ = 6.59 cm (ℓ/ℓ_*c*_ ≈ 0.83), τ_*g*_ = 20 h, and the parameters reported in Table 1. This choice of model parameters corresponds to the red dot shown in Figure 4C. For supercritical lengths (ℓ^⋆^ ≈ 6.56 cm, for such a choice of model parameters) two types of nontrivial periodic solutions emerge: **(A)** unstable pendular oscillations and **(B)** stable circular oscillatory patterns.

1. **Graviceptive case:** α = η = 0 and β > 0. In the absence of both endogenous cues (α = 0) and straightening mechanisms (η = 0), the straight equilibrium configuration becomes unstable as the plant shoot attains a critical length (see Supplementary Material, section S3.3.1). We report in Figure 4A the stability boundary for the system in terms of the characteristic time of growth τ_*g*_ and the shoot length ℓ. In the unstable region we find pendular oscillations (already observed in the planar version of the present model, Agostinelli et al., 2020b) and new three-dimensional circular trajectories, which emerge as limit cycles of the nonlinear model. In other terms, spontaneous—pendular and circular—oscillations emerge when the shoot is longer than a critical threshold, fixed all other parameters. In this case, the role of elasticity is sharp: no oscillations occur in the absence of elastic deflections due to gravity loading. Indeed, neglecting elasticity means that ℓ_*c*_ → ∞ (either by letting the rod stiffness tend to infinity or by removing the gravity loading), and in this limit the plant shoot is always stable, irrespective of τ_*g*_ (see Figure 4A).
2. **Microgravity:** α = β = 0, η > 0, and *q* = 0. As an intermediate case study, we analyze the model in microgravity conditions by discarding the twofold action of gravity: gravitropic reactions do not occur and the plant is weightless. In this way we isolate the effect of proprioceptive responses. In agreement with previous studies (Johnsson et al., 1999) we find that proprioception alone might induce spontaneous oscillations, which occur at a critical value of τ_*g*_ that is independent of the shoot length (see Figure 4B and Supplementary Material, section S3.3.2). These oscillations lack elastic deformations, as the plant is weightless. For the model parameters of Table 1, we find a critical value of approximately 3.5 h. However, this seems to be out of the range of experimental observations, thus suggesting that the persistence of oscillations in microgravity might have an endogenous origin (Johnsson et al., 2009; Kobayashi et al., 2019).
3. **Proprio-graviceptive case:** α = 0 and β, η > 0. Finally, we extend the analysis to the case in which proprioception and gravitropism coexist. We observe that—similarly to the case of microgravity but contrary to the graviceptive case—oscillations are possible whenever τ_*g*_ is less than the critical value of 3.5 h, thanks to proprioception. However, elastic deformations significantly impact the stability threshold (see Figure 4C and Supplementary Material, section S3.3.3). Indeed, this depends on ℓ/ℓ_*c*_, as found for the gravitropic case. A numerical study of the nonlinear regime reveals the occurrence of pendular and circular limit cycles in the unstable region (see Figure 5 and Video 4). Further, for a given τ_*g*_, proprioception lowers the critical length with respect to the graviceptive case, provided that the delay ${\bar{\tau }}_{r}$ and the memory time ${\bar{\tau }}_{m}$ are sufficiently large (see Supplementary Material, section S3.3.3).

These results confirm the existing hypothesis that exogenous, spontaneous oscillations might occur even in the absence of endogenous oscillators (Mugnai et al., 2015). However, experiments might lead to erroneous conclusions when compared to theoretical predictions from models that neglect elasticity, as this strongly influences the onset of oscillations.

**Figure 6:**
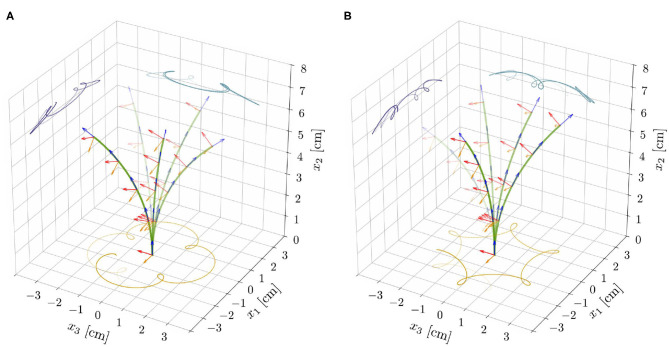
Superposition of deformed shapes and respective directors, from the reduced nonlinear rod model for ℓ = 6.565 cm, α = 0.3, and for the model parameters as reported in Table 1. Exogenous oscillations were initiated in the clockwise direction by suitable initial perturbations and epitrochoid-like **(A)** and hypotrochoid-like **(B)** patterns were obtained for concordant and discordant endogenous oscillations, respectively.

Finally, we investigate how an endogenous, time-harmonic oscillator of period τ_*e*_ affects the spontaneous oscillations that we observed in the proprio-graviceptive case. To this aim, we explored the nonlinear reduced model with α, β, η > 0, by means of the computational implementation described in Supplementary Material (section S4). In the stable region, the intrinsic oscillator dominates the dynamics and the solutions ultimately converge to motions of period τ_*e*_. On the contrary, in the unstable region, the tip traces epitrochoid-like or hypotrochoid-like patterns, depending on whether the rotational directions of the two oscillatory mechanisms are concordant or discordant, respectively (see Figure 6 and Videos 5, 6). We observe inexact periodic trochoids, as the ratio between the two periods that come into play—the one of the internal oscillator, τ_*e*_, and the one associated with the limit cycle—is typically an irrational number.

### 3.2. The Role of Plant Shoot Elongation

**Figure 7:**
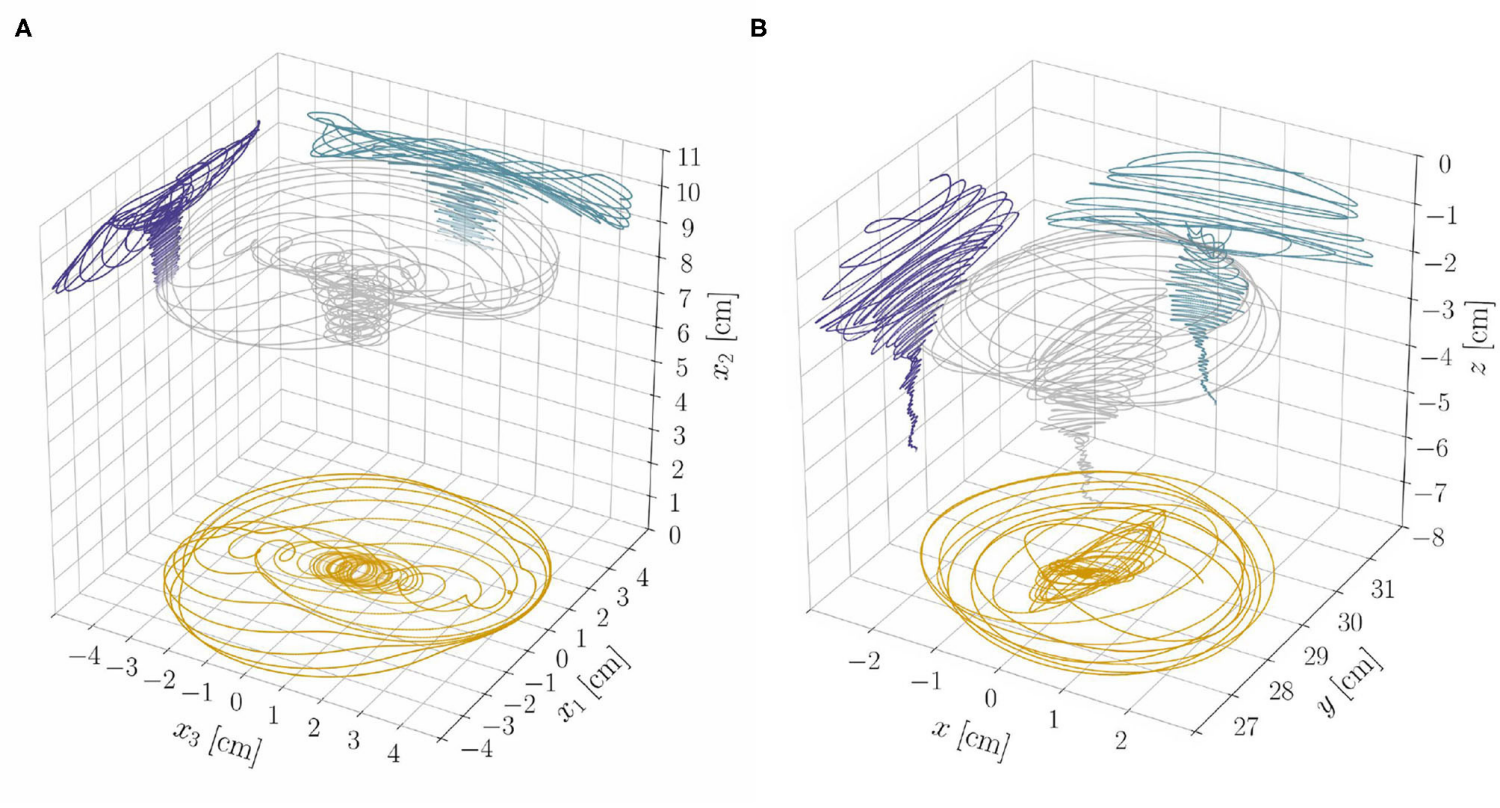
**(A)** Tip trajectory and its projections on coordinate planes from the nonlinear rod model and for the model parameters as in Video 2. Notice the progressive transition of the system from a dynamics dominated by the endogenous oscillator to one in which exogenous oscillations prevail. **(B)** Experimental results (tip trajectory and its projections on coordinate planes) from a sample of *Arabidopsis thaliana* (Col-0) are reported for qualitative comparison (see also Video 1). We refer to Supplementary Material (section S5 of Data Sheet 1 and Data Sheet 2) for more details on the experiments.

The results from the reduced model, at constant length, provide insight on the dynamics of the full model, which accounts for length changes, lignification processes, and statoliths dynamics. From section 3.1, we expect exogenous oscillations to arise once a critical length ℓ^⋆^ is attained, while all other parameters are fixed. Indeed, we find numerically (see Supplementary Material, section S4) that the relative weight of the two oscillatory mechanisms—endogenous and exogenous—changes and affects the resulting dynamics as the shoot elongates. The system gradually transitions from a dynamics mainly characterized by endogenous oscillations in the subcritical, stable regime (ℓ < ℓ^⋆^) to one in which exogenous oscillations dominate in the supercritical, unstable regime (ℓ > ℓ^⋆^). Trochoid-like patterns appear in the intermediate regime (see Figure 7A and the Videos 2, 3).

Elasticity crucially influences the transition from one dynamics to the other, which generates different nutation patterns at different stages of growth of the same plant. To assess the plausibility of these theoretical findings, as a proof of principle, we performed some qualitative experimental observations of a plant exhibiting different nutation patterns at different growth stages. We report in Figure 7B the three-dimensional tip trajectory from a specimen of *A. thaliana*, and refer to Supplementary Material (section S5) for details on the experimental procedures.

## 4. Discussion

In this study we proposed a new three-dimensional morphoelastic rod model capable of describing the motion of growing plant shoots, which accommodates an inherent straightening mechanism (proprioception), gravitropic responses, and an endogenous oscillator. This model generalizes existing approaches in the literature (Bastien et al., 2014; Bastien and Meroz, 2016; Chelakkot and Mahadevan, 2017; Chauvet et al., 2019; Meroz et al., 2019; Agostinelli et al., 2020b; Porat et al., 2020; Tsugawa et al., 2020), by simultaneously accounting for the three-dimensionality of the shoot and the elastic deformations due to gravity loading. We intended this model as a test bed for different hypotheses about circumnutations in plant shoots, but it might be informative for other biological aspects or even in the context of bioinspired soft robotics, which recently started drawing inspiration from the plant kingdom to conceive and design innovative adaptable robots (Mazzolai, 2017).

Since the first experimental observations of plant circumnutations, a long-lasting debate produced three main theories for their nature: the existence of an endogenous oscillator (Darwin, 1880), a gravitropic feedback oscillator (Gradmann, 1922), or a combination of the two (Johnsson et al., 1999; Stolarz, 2009). Previous analyses of these theories disregarded elastic deflections due to gravity loading, which however may affect in a relevant way the mechanical stability of the biological system. Indeed, we showed that in our model the onset of exogenous oscillations largely depends on elasticity, as the system suffers an instability when a loading parameter, such as the shoot length, exceeds a critical threshold. In the presence of an endogenous oscillator, the relative amplitude of the two mechanisms varies in time: endogenous oscillations prevail in the subcritical, stable regime of short shoots, while the exogenous ones dominate the supercritical, unstable regime of long shoots; in the intermediate, transient regime, the two competing oscillations can reproduce trochoid-like patterns (for which Schuster and Engelmann, 1997 provided experimental evidence). Interestingly, Darwin (1880) described a similar dynamics: “[…] *climbing plants whilst young circumnutate in the ordinary manner, but as soon as the stem has grown to a certain height, which is different for different species, it elongates rapidly, and now the amplitude of the circumnutating movement is immensely increased, evidently to favor the stem catching hold of a support* […].” As a further proof of concept, we observed experimentally the transitions between different patterns and amplitude on the primary inflorescence of a specimen of *A. thaliana* Col-0 growing under continuous light (see Figure 7B and Video 1).

The reality of plant circumnutations is more complex than our simple modeling assumptions, and our theoretical predictions require a thorough quantitative assessment in comparison with experimental observations. However, we believe that this study provides the possibility to reinterpret the “two-oscillator hypothesis” and the vast existing experimental literature from a renewed perspective. Our results suggest the potential role of elasticity in circumnutations and provide a minimal mechanism/hypothesis that might account for the variability of the patterns and amplitudes of observed nutations, not only across different plant species and specimens but also at different stages of growth of the same plant.

## Data Availability Statement

The experimental data presented in this article are accessible as Supplementary Material at: https://www.frontiersin.org/articles/10.3389/fpls.2021.608005/full#supplementary-material. For simulations, the Python codes based on the FEniCS Project Version 2019.1.0 (Logg et al., 2012) are available at: https://github.com/mathLab/MorphoelasticRod.

## Author Contributions

All authors conceived the study, contributed to the theoretical analysis, and wrote the manuscript. GN and DA performed the experiments. DA analyzed the data, wrote the code, and carried out numerical computations. All authors gave final approval for publication and agree to be held accountable for the work performed therein.

## Conflict of Interest

The authors declare that the research was conducted in the absence of any commercial or financial relationships that could be construed as a potential conflict of interest.

## Supplementary Material

Data Sheet 1 Five Supplementary sections: Growing morphoelastic rods, the statoliths avalanche dynamics, stability analyses, computational model, and experimental materials and methods.

Data Sheet 2 Experimental Data. We provide 4 csv files (data_fig1A.csv, data_fig1B.csv, data_fig1C.csv, and data_video.csv) with the tip trajectories shown in this article (Figures 1, 7 and Video 1), as reconstructed from the experiments (see Supplementary Material, section S5).

Video 1 Experiment and data analysis. This video shows the tracking and reconstruction of a flower of the primary inflorescence in a 29-day-old sample of *Arabidopsis thaliana* (ecotype Col-0) grown under normal gravity conditions (1 g) and continuous light at the SAMBA laboratory of SISSA. The images were acquired by using two digital cameras, namely, two acA4024-29uc c mount cameras from Basler, both of which were equipped with an objective m0824-mpw2 from Computar. The points (black dots) were tracked on the stereo pair of images, which were calibrated to reconstruct the 3D position by exploiting the Computer Vision Toolbox in MATLAB R2019b. The colored lines, from blue to red for increasing time, are obtained by moving averaging over ten detected positions. The red dot indicates the base of the plant.

Video 2 A growing shoot. Computational results from the full model (S3.1) for ℓ_0_ = 6.8 cm, ℓ_*g*_ = 7 cm, α = 0.2, β = 0.8, η = 20, τ_*a*_ = 2 min, $\tau_{m}=\tau_{r}={\bar{\tau }}_{m}={\bar{\tau }}_{r}=12$ min, τ_*e*_ = 24 min, τ_*g*_ = 40 h, τ_ℓ_ = 6 d, *r* = 0.5 mm, ρ = 10^3^ Kg m^−3^, $E_{0}=10^{7}$ Pa, *E*_1_ = 200*E*_0_, and ν = 0.5. We notice that the critical length for the stability of the corresponding reduced model (S3.5) is given by ℓ^⋆^ ≈ 7.24 cm for the chosen model parameters. Here black arrows denote the statoliths local orientations (**h**), while the other three are the directors (**d**_1_, **d**_2_ and **d**_3_). In the beginning we observe only endogenous oscillations with gravitropic and proprioceptive responses. In the intermediate regime endogenous and exogenous oscillations are comparable and give rise to trochoid-like patterns. In the end, the second ones dominate the oscillatory movements.

Video 3 A growing shoot. Same as in Video 2 but for α = 0.01. Differently from Video 2, intrinsic cues have little influence and the growing shoot tends to circular patterns passing by elliptic trajectories.

Video 4 Limit cycles. Computational results from the reduced model (S3.5) but equipped with the statolith avalanche dynamics (6). Model parameters are ℓ = 6.59 cm, α = 0, β = 0.8, η = 20, $\tau_{m}=\tau_{r}={\bar{\tau }}_{m}={\bar{\tau }}_{r}=12$ min, τ_*a*_ = 2 min, τ_*g*_ = 20 h, *r* = 0.5 mm, ρ = 10^3^ Kg m^−3^, *E* = 10^7^ Pa, and ν = 0.5. Black arrows denote the statoliths local orientations (**h**), while the other three are the directors (**d**_1_, **d**_2_ and **d**_3_). We initially perturb the model with an apical load ϵsin(π*t*/12)(2**e**_1_ + **e**_3_) where ϵ = 10^−5^ N for *t* ∈ [0, 12] min, and {**e_1_**, **e_2_**, **e_3_**} is a fixed orthonormal basis. Upon such a perturbation, the solution tends to pendular oscillations until an orthogonal apical load ϵsin[π(*t*−1800)/12](**e**_1_−2**e**_3_) is applied for *t* ∈ [1800, 1812] min. Then the solution tends to the stable circular limit cycle by describing elliptic spirals.

Video 5 Epitrochoid-like patterns. Same model as in Video 4, but for α = 0.3, τ_*e*_ = 20 min and ℓ = 6.565 cm. In order to speed up the convergence to periodic oscillations, we introduced some perturbations as apical loads at different time intervals (not shown).

Video 6 Hypotrochoid-like patterns. Same as in Video 5 but with opposite angular velocity for the internal oscillator.
