# Supplementary material for: Nutations in Plant Shoots: Endogenous and Exogenous Factors in the Presence of Mechanical Deformations
Source: Front Plant Sci. 2021 Mar 23;12:608005. doi: 10.3389/fpls.2021.608005 (PMC8023405; doi:10.3389/fpls.2021.608005)
Supplement: Data Sheet 1 — Five Supplementary sections: Growing morphoelastic rods, the statoliths avalanche dynamics, stability analyses, computational model, and experimental materials and methods. [file Data_Sheet_1.pdf]

frontiers

Supplementary Material

CONTENTS

S1 Growing morphoelastic rods 1
S1.1 Kinematics: Initial, virtual and current configuration 1
S1.2 Mechanics and constitutive assumptions 2
S1.3 Subapical growth 3
S1.4 Differential growth and evolution laws 4
S2 The statoliths avalanche dynamics 10
S3 Stability analyses 12
S3.1 Summary of the model for growing plant shoots 12
S3.2 Representation in terms of Euler angles 12
S3.3 Analysis of the reduced model for short times 14
S4 Computational model 23
S5 Experimental materials and methods 26

S1 GROWING MORPHOELASTIC RODS

Building on the theory of morphoelasticity, we propose a 3D rod model to describe elongating, slender plant organs. We do so under the key assumption that the time-scale for mechanical equilibrium is much shorter than any biological time-scale of the plant.

S1.1 Kinematics: Initial, virtual and current configuration

Consider an Euclidean space $\mathbb{E}^3$ with a fixed right-handed orthonormal basis $\{\mathbf{e}_1, \mathbf{e}_2, \mathbf{e}_3\}$ and define three different configurations of the rod:

- An initial reference configuration $\mathcal{B}_0$ given by a rod having axis $\mathbf{p}_0(S)$ and material cross sections characterized by the orthonormal directors $\{\mathbf{d}_1^0(S), \mathbf{d}_2^0(S), \mathbf{d}_3^0(S) := \mathbf{d}_1^0(S) \times \mathbf{d}_2^0(S)\}$ where $S \in [0, \ell_0]$ is the arc length material parameter describing the distance from the base;
- A virtual reference configuration $\mathcal{B}_v(t)$ defined as the unstressed realization of the rod at time t, having axis $\mathbf{p}_v(s_v, t)$ and orthonormal directors $\{\mathbf{d}_1^v(s_v, t), \mathbf{d}_2^v(s_v, t), \mathbf{d}_3^v(s_v, t) := \mathbf{d}_1^v(s_v, t) \times \mathbf{d}_2^v(s_v, t)\}$ where $s_v \in [0, \ell_v(t)]$ is the arc length coordinate;
- A current configuration $\mathcal{B}(t)$ which is the actual shape of the rod at time t, taking into account deflection from mechanical loads and boundary conditions. Such a rod is defined by the space curve $\mathbf{p}(s, t)$ equipped with the triple of right-handed orthonormal directors $\{\mathbf{d}_1(s, t), \mathbf{d}_2(s, t), \mathbf{d}_3(s, t) := \mathbf{d}_1(s, t) \times \mathbf{d}_2(s, t)\}$ where $s \in [0, \ell(t)]$ is the arc length parameter.

In particular, we choose the initial reference configuration as the virtual configuration at time $t = 0$, namely, $\ell_0 := \ell_v(0)$ and $\mathbf{d}_j^0(S) := \mathbf{d}_j(S, 0)$ for all $S \in [0, \ell_0]$.

Since the parameter S is a material coordinate for both the virtual and the current configuration, we define the respective motions, namely,

$$s_v(\cdot, t) : [0, \ell_0] \to [0, \ell_v(t)] \quad \text{and} \quad s(\cdot, t) : [0, \ell_0] \to [0, \ell(t)] \,, \tag{S1.1}$$

and we denote their inverse functions by the same symbol $S(\cdot, t)$. Moreover, in order to simplify the notation, we use the same symbol to denote material and spatial descriptions of any given field. Therefore

any field defined on one of the three configurations can be evaluated at each of the other ones, by means of an implicit composition of functions. For example, given a Lagrangian (or material) field $f(S,t) : [0, \ell_0] \to \mathbb{R}$, the associated Eulerian (or spatial) field is simply denoted by $f(s,t) := f(S(s,t),t) : [0, \ell(t)] \to \mathbb{R}$.

In the following we use a superimposed dot to denote the material time derivatives of any spatial vector or scalar field.

In this framework we introduce the *full axial stretch* as

$$\lambda(S,t) := \frac{\partial s(S,t)}{\partial S}, \tag{S1.2}$$

which can be decomposed in the product $\lambda(S,t) = \sigma(s_v(S,t),t)\gamma(S,t)$ where

$$\sigma(s_v,t) := \frac{\partial s(s_v,t)}{\partial s_v} \quad \text{and} \quad \gamma(S,t) := \frac{\partial s_v(S,t)}{\partial S}, \tag{S1.3}$$

are the *elastic stretch* and the *growth stretch*, respectively. Then, we define the *true strains*

$$\varepsilon(S,t) := \ln \lambda(S,t) \quad \text{and} \quad \varepsilon_v(S,t) := \ln \gamma(S,t), \tag{S1.4}$$

which will turn out to be crucial growth quantifier.

From classical rod theory (Antman, 2005), we know that there exist vector-valued functions $\mathbf{u}(s,t)$, called *Darboux vector*, and $\mathbf{w}(s,t)$, called *spin*, such that

$$\partial_{s_v}\mathbf{d}_j = \mathbf{u} \times \mathbf{d}_j \quad \text{and} \quad \partial_t \mathbf{d}_j = \mathbf{w} \times \mathbf{d}_j. \tag{S1.5}$$

As for the components $u_j := \mathbf{u} \cdot \mathbf{d}_j$, these are referred to as *flexural strains* for $j = 1, 2$ and *torsional strain* for $j = 3$. In a similar manner, the directors of the virtual configuration define the *spontaneous Darboux vector*, $\mathbf{u}^\star$, and the *spontaneous spin*, $\mathbf{w}^\star$, *i.e.*,

$$\partial_{s_v}\mathbf{d}_j^v = \mathbf{u}^\star \times \mathbf{d}_j^v \quad \text{and} \quad \partial_t \mathbf{d}_j^v = \mathbf{w}^\star \times \mathbf{d}_j^v, \tag{S1.6}$$

and the components $u_j^\star := \mathbf{u}^\star \cdot \mathbf{d}_j$ are called *spontaneous strains*.

S1.2 Mechanics and constitutive assumptions

Under the quasi-static assumption we impose the static equilibrium in the virtual reference configuration at all times, such that

$$\mathbf{n}'(s_v,t) + \mathbf{f}(s_v,t) = \mathbf{0}, \tag{S1.7a}$$

$$\mathbf{m}'(s_v,t) + \mathbf{p}'(s_v,t) \times \mathbf{n}(s_v,t) + \mathbf{l}(s_v,t) = \mathbf{0}, \tag{S1.7b}$$

where a prime denotes differentiation with respect to s_v, $\mathbf{n}$ and $\mathbf{m}$ are the resultant contact force and contact couple, whereas $\mathbf{f}$ and $\mathbf{l}$ are the body force and couple per unit virtual reference length, respectively. Determination of the current configuration $\mathcal{B}(t)$ can be achieved by solving equations (S1.7) combined with a suitable constitutive model and appropriate boundary conditions.

For plant organs, a reasonable assumption is to treat them as unshearable ($\mathbf{d}_3 = \partial_s \mathbf{p}$) and elastically inextensible rods, such that $\sigma = 1$ and $s = s_v$, and characterized by a quadratic strain-energy function

defined by a diagonal stiffness matrix $\mathbf{K}$, such that the constitutive law reads

$$\mathbf{m} = \sum_j K_j \left(u_j - u_j^\star\right) \mathbf{d}_j, \tag{S1.8}$$

where K_j denotes the j-th diagonal component of $\mathbf{K}$. More specifically, in this study we assume rods of circular cross section of radius r, such that $K_1 = K_2 = EI$, where E is the Young's modulus and $I = \pi r^4/4$ is the second moment of inertia, and $K_3 = \mu J$ where $J = 2I$ and $\mu = 2E(1+\nu)$ is the shear modulus determined by the Poisson's ratio ν. In passing, we notice that such a modeling assumption might be refined by considering elliptic cross sections, which provide more accurate descriptions of some plant organs (Paul-Victor and Rowe, 2011).

In the absence of external loads and couples, *i.e.*, for $\mathbf{f} = \mathbf{0}$ and $\mathbf{l} = \mathbf{0}$, equations (S1.7) lead to $\mathbf{n} = \mathbf{0}$ and $\mathbf{m} = \mathbf{0}$, in which case the visible strains coincide with the spontaneous strains of the virtual configuration, $\mathcal{B}_v(t)$. Therefore, in this special case we recover the kinematic model recently proposed by Porat et al. (2020).

S1.3 Subapical growth

In order to describe the primary growth of plant organs, Erickson and Sax (1956) introduced the notion of *relative elemental growth rate* (REGR) or *relative elongation rate* (RER), which in our notation is the material gradient of the Lagrangian velocity field $v_v(S,t) := \partial_t s_v(S,t)$, namely,

$$\mathrm{REGR}(s_v,t) := \operatorname{grad} v_v(s_v,t) = \frac{\partial v_v(s_v,t)}{\partial s_v} = \frac{\partial}{\partial s_v}\left(\left.\frac{\partial s_v}{\partial t}\right|_{S(s_v,t)} \right). \tag{S1.9}$$

Such a quantity is related to the deformation gradient, $F = \gamma$, by means of the relationship $\operatorname{grad} v_v = \dot{F}F^{-1}$, which explicitly reads

$$\frac{\partial}{\partial s_v}\left(\left.\frac{\partial s_v}{\partial t}\right|_{S(s_v,t)} \right) = \left.\frac{\partial}{\partial t}\left(\frac{\partial s_v}{\partial S}\right)\right|_{S(s_v,t)} \cdot \frac{\partial S}{\partial s_v} = \left.\left(\frac{1}{\gamma}\frac{\partial \gamma}{\partial t}\right)\right|_{S(s_v,t)}, \tag{S1.10}$$

thus yielding

$$\mathrm{REGR}(s_v,t) = \left.\left(\frac{1}{\gamma}\frac{\partial \gamma}{\partial t}\right)\right|_{S(s_v,t)} =: \dot{\varepsilon}_v(s_v,t), \tag{S1.11}$$

where dots denote material time derivatives.

Therefore, subapical growth is prescribed by the following coupled problems,

$$\frac{\partial s_v}{\partial S}(S,t) = \gamma(S,t) \quad \text{with} \quad s_v(0,t) = 0, \tag{S1.12a}$$

$$\frac{1}{\gamma(S,t)}\frac{\partial \gamma}{\partial t}(S,t) = \mathrm{REGR}(S,t) \quad \text{with} \quad \gamma(S,0) = 1, \tag{S1.12b}$$

for $S \in [0, \ell_0]$ and $t \geq 0$, which can be integrated to get

$$s_v(S,t) = \int_0^S e^{\int_0^t \mathrm{REGR}(\zeta,\tau)\,\mathrm{d}\tau}\,\mathrm{d}\zeta. \tag{S1.13}$$

Following previous studies (Bastien et al., 2014; Chelakkot and Mahadevan, 2017), among the possible choices, we consider a piecewise constant growth function, namely, $\mathrm{REGR}(S,t) = k\,\mathrm{H}(S - P(t))$ where k is a positive constant, H is the Heaviside function, and $P(t) := S(\ell_v(t) - \ell_g, t)$ denotes the material point that exits the growth zone at time t. By means of equation (S1.13),

$$\begin{aligned}\ell_v(t) :=& s_v(\ell_0, t) = \int_0^{\ell_0} e^{\int_0^t k\,\mathrm{H}(\zeta - P(\tau))\,\mathrm{d}\tau}\,\mathrm{d}\zeta = \int_0^{P(t)} e^{\int_0^t k\,\mathrm{H}(\zeta - P(\tau))\,\mathrm{d}\tau}\,\mathrm{d}\zeta + \int_{P(t)}^{\ell_0} e^{kt}\,\mathrm{d}\zeta \\ =& s_v(P(t), t) + [\ell_0 - P(t)]\,e^{kt} = \ell_v(t) - \ell_g + [\ell_0 - P(t)]\,e^{kt},\end{aligned} \tag{S1.14}$$

whence

$$P(t) = \ell_0 - \ell_g e^{-kt}, \tag{S1.15}$$

whose inverse is

$$t^\star(S) = \frac{1}{k}\ln\left(\frac{\ell_g}{\ell_0 - S}\right) \quad \text{for } S \geq \ell_0 - \ell_g, \tag{S1.16}$$

that is the instant of time at which the cell initially located at S stops elongating. We notice that $\lim_{S\to\ell_0} t^\star(S) = \infty$, namely, the subapical region is never going to stop growing. By combining equations (S1.15)-(S1.16) with equation (S1.13), we arrive at

$$\begin{aligned} s_v(S,t) =& \left[1 - \mathrm{H}\left(S - (\ell_0 - \ell_g)\right)\right] S \\ &+ \mathrm{H}\left(S - (\ell_0 - \ell_g)\right) \Big\{ \mathrm{H}(t - t^\star(S)) \left[\max\{\ell_0, \ell_g\} + \ell_g k \min\{t^\star(S), t^\star(S) - t^\star(0)\} - \ell_g\right] \\ &+ [1 - \mathrm{H}(t - t^\star(S))] \Big[-(\ell_0 - S)e^{kt} + [1 - \mathrm{H}(t - t^\star(0))]\ell_0 e^{kt} \\ &+ \mathrm{H}(t - t^\star(0)) \left[\max\{\ell_0, \ell_g\} + \ell_g k \min\{t, t - t^\star(0)\}\right] \Big] \Big\}, \end{aligned} \tag{S1.17}$$

whence

$$\ell_v(t) := s_v(\ell_0, t) = \begin{cases} \ell_0 e^{kt} & \text{if } t \leq t^\star(0), \\ \max\{\ell_0, \ell_g\} + \ell_g k\,(t - \max\{0, t^\star(0)\}) & \text{if } t > t^\star(0). \end{cases} \tag{S1.18}$$

Finally, equation (S1.17) can be rewritten in the following compact form

$$s_v(S,t) = \begin{cases} S & \text{if } S \leq \ell_0 - \ell_g, \\ \ell_v(t^\star(S)) - \ell_g & \text{if } S > \ell_0 - \ell_g \text{ and } t \geq t^\star(S), \\ \ell_v(t) - (\ell_0 - S)e^{kt} & \text{if } S > \ell_0 - \ell_g \text{ and } t < t^\star(S), \end{cases} \tag{S1.19}$$

which is shown in Fig. 2 of the main text.

S1.4 Differential growth and evolution laws

The shape of growing plant roots and shoots evolves and adapts by responding to a variety of stimuli. The main morphing mechanism consists in a spatially nonhomogeneous growth rate of the cross section, called *differential growth*. In this section we determine the relationship between differential growth and strain rates.

First of all, we use equation (S1.11) to extend the notion of relative elemental growth rate to any point lying on the circular cross section s_v of the virtual configuration at time t. We parameterize the rod by means of the spatial coordinates (x, y) in the local basis $\{\mathbf{d}_1^v(s_v, t), \mathbf{d}_2^v(s_v, t)\}$, namely,

$$\mathbf{p}_v(s_v, t; x, y) := \mathbf{p}_v(s_v, t) + x\, \mathbf{d}_1^v(s_v, t) + y\, \mathbf{d}_2^v(s_v, t). \tag{S1.20}$$

Then the length of the material fiber passing through point (x, y) of the cross section s_v and extending from the rod's base to that point, can be written as

$$\begin{aligned} \ell_v(s_v, t; x, y) &:= \int_0^{s_v} \left(\partial_{s_v}\mathbf{p}_v(\zeta, t; x, y) \cdot \partial_{s_v}\mathbf{p}_v(\zeta, t; x, y)\right)^{\frac{1}{2}} \mathrm{d}\zeta \\ &= \int_0^{s_v} \left\{[1 + u_1^\star y - u_2^\star x]^2 + (x^2 + y^2)\, {u_3^\star}^2\right\}^{\frac{1}{2}} \mathrm{d}\zeta. \end{aligned} \tag{S1.21}$$

Equation (S1.21) follows by using the kinematic relationships $\partial_{s_v}\mathbf{p}_v = \mathbf{d}_3^v$ and $\partial_{s_v}\mathbf{d}_i^v = \mathbf{u}^\star \times \mathbf{d}_i^v\ \forall\, i$, where $\mathbf{u}^\star = \sum_j u_j^\star \mathbf{d}_j^v$ is the spontaneous Darboux vector. Then the growth stretch at (x, y) is given by

$$\begin{aligned} \gamma(s_v, t; x, y) :=& \left.\frac{\partial \ell_v(s_v, t; x, y)}{\partial S}\right|_{S=S(s_v,t)} = \left.\frac{\partial \ell_v(s_v, t; x, y)}{\partial s_v}\frac{\partial s_v}{\partial S}\right|_{S=S(s_v,t)} \\ =& \gamma(s_v, t) \left.\left\{[1 + u_1^\star y - u_2^\star x]^2 + \left(x^2 + y^2\right) {u_3^\star}^2\right\}^{\frac{1}{2}}\right|_{(s_v,t)}, \end{aligned} \tag{S1.22}$$

so that the true strain rate reads

$$\dot{\varepsilon}_v(s_v, t; x, y) = \frac{\dot{\gamma}}{\gamma}(s_v, t; x, y) = \dot{\varepsilon}_v(s_v, t) + \frac{\left[(1 + u_1^\star y - u_2^\star x)\,(\dot{u}_1^\star y - \dot{u}_2^\star x) + u_3^\star \dot{u}_3^\star \left(x^2 + y^2\right)\right]}{(1 + u_1^\star y - u_2^\star x)^2 + {u_3^\star}^2\,(x^2 + y^2)}. \tag{S1.23}$$

By differentiating expression (S1.23) with respect to x and y, we get

$$\begin{aligned} \partial_x \dot{\varepsilon}_v(s_v, t; x, y) =& \frac{\left[-\dot{u}_2^\star - (u_1^\star \dot{u}_2^\star + u_2^\star \dot{u}_1^\star)\, y + 2\,(u_2^\star \dot{u}_2^\star + u_3^\star \dot{u}_3^\star)\, x\right] \left[(1 + u_1^\star y - u_2^\star x)^2 + {u_3^\star}^2 \left(x^2 + y^2\right)\right]}{\left[(1 + u_1^\star y - u_2^\star x)^2 + {u_3^\star}^2\,(x^2 + y^2)\right]^2} \\ &- \frac{\left[(1 + u_1^\star y - u_2^\star x)\,(\dot{u}_1^\star y - \dot{u}_2^\star x) + u_3^\star \dot{u}_3^\star \left(x^2 + y^2\right)\right] \left[-2u_2^\star\,(1 + u_1^\star y - u_2^\star x) + 2{u_3^\star}^2 x\right]}{\left[(1 + u_1^\star y - u_2^\star x)^2 + {u_3^\star}^2\,(x^2 + y^2)\right]^2}, \end{aligned} \tag{S1.24}$$

and

$$\partial_y \dot{\varepsilon}_v(s_v, t; x, y) = \frac{\left[\dot{u}_1^\star - (u_1^\star \dot{u}_2^\star + u_2^\star \dot{u}_1^\star)\, x + 2\,(u_1^\star \dot{u}_1^\star + u_3^\star \dot{u}_3^\star)\, y\right] \left[(1 + u_1^\star y - u_2^\star x)^2 + {u_3^\star}^2 \left(x^2 + y^2\right)\right]}{\left[(1 + u_1^\star y - u_2^\star x)^2 + {u_3^\star}^2\,(x^2 + y^2)\right]^2}$$

$$-\frac{\left[(1+u_1^\star y-u_2^\star x)\left(\dot{u}_1^\star y-\dot{u}_2^\star x\right)+u_3^\star \dot{u}_3^\star\left(x^2+y^2\right)\right]\left[2u_1^\star\left(1+u_1^\star y-u_2^\star x\right)+2{u_3^\star}^2 y\right]}{\left[(1+u_1^\star y-u_2^\star x)^2+{u_3^\star}^2\left(x^2+y^2\right)\right]^2}, \tag{S1.25}$$

respectively. Therefore, by Taylor expanding (S1.23) about the cross section center $(0,0)$, we arrive at

$$\dot{\varepsilon}_v(s_v,t;x,y)=\dot{\varepsilon}_v(s_v,t)+\boldsymbol{\delta}_v(s_v,t)\cdot\left(x\,\mathbf{d}_1^v(s_v,t)+y\,\mathbf{d}_2^v(s_v,t)\right)+o(\sqrt{x^2+y^2}), \tag{S1.26}$$

where x and y are the coordinates of the point in the local basis $\{\mathbf{d}_1^v,\mathbf{d}_2^v\}$, and

$$\boldsymbol{\delta}_v(s_v,t):=\nabla\dot{\varepsilon}_v(s_v,t;0,0)=\dot{u}_1^\star(s_v,t)\mathbf{d}_2^v(s_v,t)-\dot{u}_2^\star(s_v,t)\mathbf{d}_1^v(s_v,t) \tag{S1.27}$$

is the *growth gradient* on the virtual cross section s_v at time t. Hence the corresponding growth gradient in the current configuration is given by

$$\boldsymbol{\delta}(s,t)=\dot{u}_1^\star(s,t)\mathbf{d}_2(s,t)-\dot{u}_2^\star(s,t)\mathbf{d}_1(s,t), \tag{S1.28}$$

which is orthogonal to the axis of bending, see Fig. S1A.

Remark S1.1. Prescribing the *growth gradient* $\boldsymbol{\delta}_v:=\nabla\dot{\varepsilon}_v(s_v,t;0,0)$ is equivalent to the approaches taken in previous studies (Bastien et al., 2014; Bastien and Meroz, 2016; Porat et al., 2020), which involve a notion of *differential growth* $\mathrm{DG}(s_v,t;\vartheta)$ introduced as a means to compare strains at diametrically opposite sides of the circular cross section. Indeed, by passing to the polar coordinates (ρ,ϑ), such that $(x,y)=(\rho\cos\vartheta,\rho\sin\vartheta)$, equation (S1.23) reads

$$\dot{\varepsilon}_v(s_v,t;\rho,\vartheta)=\dot{\varepsilon}_v(s_v,t)+\frac{\left[(1+u_1^\star\rho\sin\vartheta-u_2^\star\rho\cos\vartheta)\left(\dot{u}_1^\star\rho\sin\vartheta-\dot{u}_2^\star\rho\cos\vartheta\right)+u_3^\star\dot{u}_3^\star\rho^2\right]}{(1+u_1^\star\rho\sin\vartheta-u_2^\star\rho\cos\vartheta)^2+{u_3^\star}^2\rho^2}, \tag{S1.29}$$

and then, the differential growth can be defined as

$$\mathrm{DG}(s_v,t;\vartheta):=\frac{\dot{\varepsilon}_v(s_v,t;r,\vartheta)-\dot{\varepsilon}_v(s_v,t;r,\vartheta+\pi)}{\dot{\varepsilon}_v(s_v,t;r,\vartheta)+\dot{\varepsilon}_v(s_v,t;r,\vartheta+\pi)} \tag{S1.30}$$

$$=\frac{(\dot{a}-\dot{b})\left(A+(b-a)B\right)-c\dot{c}B}{\dot{\varepsilon}_v(s_v,t)\left(A^2-B^2\right)+(\dot{a}-\dot{b})\left((a-b)A-B\right)+c\dot{c}}, \tag{S1.31}$$

or

$$\mathrm{DG}(s_v,t;\vartheta):=\frac{\dot{\varepsilon}_v(s_v,t;r,\vartheta)-\dot{\varepsilon}_v(s_v,t;r,\vartheta+\pi)}{2\dot{\varepsilon}_v(s_v,t)}=\frac{(\dot{a}-\dot{b})\left(A+(b-a)B\right)-c\dot{c}B}{\dot{\varepsilon}_v(s_v,t)\left(A^2-B^2\right)}, \tag{S1.32}$$

where $a:=u_1^\star r\sin\vartheta$, $b:=u_2^\star r\cos\vartheta$, $c:=u_3^\star r$, $A:=1+(a-b)^2+c^2$ and $B:=2(a-b)$. In both cases, by assuming that $ru_j^\star\ll 1$, the differential growth DG can be approximated as

$$\mathrm{DG}(s_v,t;\vartheta)\simeq\frac{r}{\dot{\varepsilon}_v}(\dot{u}_1^\star\sin\vartheta-\dot{u}_2^\star\cos\vartheta)=\frac{r}{\dot{\varepsilon}_v}\left(\dot{u}_1^\star\mathbf{d}_2^v-\dot{u}_2^\star\mathbf{d}_1^v\right)\cdot\mathbf{a}(s_v,t;\vartheta), \tag{S1.33}$$

where $\mathbf{a}(s_v, t; \vartheta) := \cos\vartheta \mathbf{d}_1^v(s_v, t) + \sin\vartheta \mathbf{d}_2^v(s_v, t)$. Therefore, by comparing expressions (S1.26) and (S1.33), we deduce that prescribing the *differential growth* $\mathrm{DG}(s_v, t; \vartheta)$ for all $\vartheta \in [0, 2\pi)$ is equivalent to prescribe the *growth gradient* $\boldsymbol{\delta}_v := \nabla\dot{\varepsilon}_v(s_v, t; 0, 0)$, as introduced by means of equation (S1.27).

Equation (S1.28) reveals the connection between differential growth and spontaneous strain rates. Indeed when the growth rate of the cross section is affine, or the organ radius is small enough to justify a linearization, the prescription of the growth gradient $\boldsymbol{\delta}$ results in the evolution laws for the spontaneous flexural strains $u_1^\star$ and $u_2^\star$. We observe that the contribution of the torsional strain $u_3^\star$ to the growth gradient is negligible. Nevertheless, it could play a crucial role in other growth mechanisms, such as that observed in twining plants.

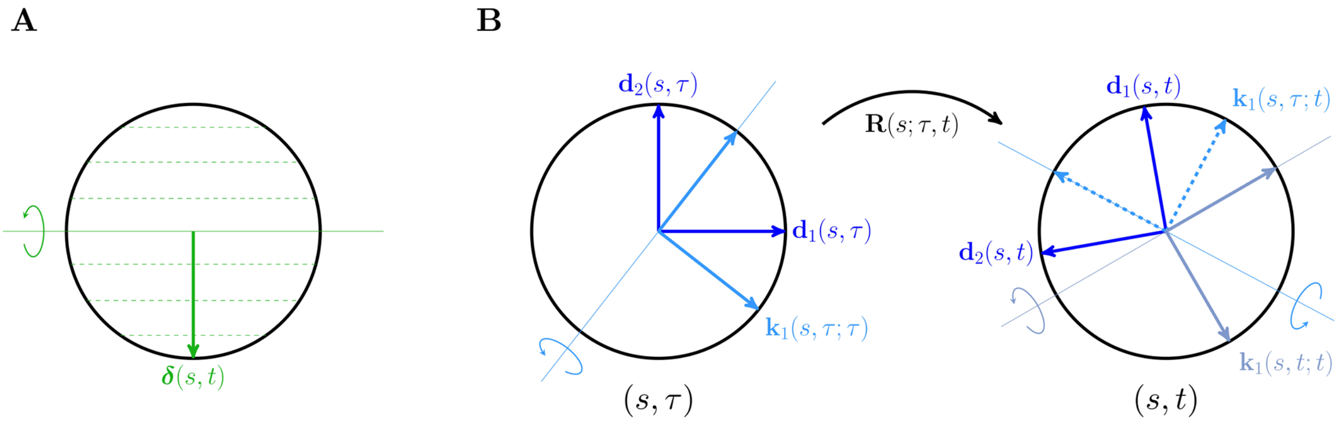

Figure S1. **(A)** Level curves of an affine strain rate having gradient $\boldsymbol{\delta}$, on the cross section (s,t); the axis perpendicular to $\boldsymbol{\delta}$ is the one about which bending occurs as due to differential growth. **(B)** Time evolution of the material cross section. $\mathbf{R}(s;\tau,t)$ is the rotation mapping $\mathbf{d}_j(s,\tau)$ into $\mathbf{d}_j(s,t)$, whereas $\mathbf{k}_1(s,\tau;t)$ denotes the contribution to the growth gradient at time t due to a stimulus sensed at time τ.

In the presence of n different stimuli, we assume a weighted average of their respective growth gradients defined on the current cross section. In other terms, the overall growth gradient for a circular cross section of radius r is determined by

$$\boldsymbol{\delta} = \sum_{j=1}^{n} \boldsymbol{\delta}^j \quad \text{with} \quad \boldsymbol{\delta}^j(s,t) := \frac{\dot{\varepsilon}_v(s,t)}{r} \int_{-\infty}^{t} \mu_j(s,\tau;t)\mathbf{k}_1^j(s,\tau;t)\,\mathrm{d}\tau, \tag{S1.34}$$

where $\mu_j(s,\tau;t)\mathbf{k}_1^j(s,\tau;t)$ is the vector on the *current* cross section that defines the contribution to the growth gradient from the j-th stimulus sensed at time τ. We project equation (S1.34) on the local basis $\{\mathbf{d}_1, \mathbf{d}_2\}$ to get

$$\dot{u}_1^\star(s,t) = \frac{\dot{\varepsilon}_v(s,t)}{r} \sum_{j=1}^{n} \int_{-\infty}^{t} \mu_j(s,\tau;t)\mathbf{k}_1^j(s,\tau;\tau) \cdot \mathbf{d}_2(s,\tau)\,\mathrm{d}\tau, \tag{S1.35a}$$

$$\dot{u}_2^\star(s,t) = -\frac{\dot{\varepsilon}_v(s,t)}{r} \sum_{j=1}^{n} \int_{-\infty}^{t} \mu_j(s,\tau;t)\mathbf{k}_1^j(s,\tau;\tau) \cdot \mathbf{d}_1(s,\tau)\,\mathrm{d}\tau, \tag{S1.35b}$$

where we have used the fact that the contribution to growth sensed at a certain time τ is fixed in the frame of the directors, namely, $\mathbf{k}_1(s,\tau;t) = \mathbf{R}(s;\tau,t)\mathbf{k}_1(s,\tau;\tau)$ where $\mathbf{R}(s;\tau,t)$ is the rotation that maps $\mathbf{d}_j(s,\tau)$ into $\mathbf{d}_j(s,t)$, as illustrated in Fig. S1B.

In this study we use equations (S1.35) to describe a response of a material cross section to a stimulus sensed at the very same location. However, they might be adapted to the case of nonlocal responses, as it occurs for gravitropic reactions in plant roots (Nakamura et al., 2019). Moreover, these expressions allow to include memory and delay effects, as done in recent studies (Chauvet et al., 2019; Meroz et al., 2019), and the instantaneous models are recovered as special cases by choosing the Dirac delta as response function.

In the following we discuss the plant response to different stimuli: Endogenous prescription (internal oscillators), reorientation to align the organ axis with a given vector (gravitropism), and straightening mechanism (proprioception).

S1.4.1 Endogenous cues

Inspired by the Darwinian concept of internal oscillator, we implement an endogenous driver for the differential growth mechanism. The simplest approach is to assign a spatially uniform time-harmonic oscillator, such that

$$\boldsymbol{\delta}^e(s,t) = \frac{\alpha}{r}\dot{\varepsilon}_v(s,t)\left[\cos\left(2\pi t/\tau_e\right)\mathbf{d}_1(s,t) + \sin\left(2\pi t/\tau_e\right)\mathbf{d}_2(s,t)\right], \tag{S1.36}$$

where τ_e is the period of endogenous oscillations and α is a dimensionless sensitivity constant.

S1.4.2 Reorientation under directional cues (Gravitropism)

Any vector stimulus s sensed in the current configuration, towards (or away from) which the plant organ aligns via differential growth (*e.g.*, gravitropism and phototropism for a far light source), contributes to growth gradient via its projection on the plane $(\mathbf{d}_1, \mathbf{d}_2)$, such that

$$\mathbf{k}_1^s = \frac{\mathbf{s} - (\mathbf{d}_3\cdot\mathbf{s})\mathbf{d}_3}{||\mathbf{s} - (\mathbf{d}_3\cdot\mathbf{s})\mathbf{d}_3||}. \tag{S1.37}$$

Notice that the direction of null differential growth, about which the organ bends, is given by $\mathbf{k}_2^s := \mathbf{d}_3 \times \mathbf{k}_1^s$. Then the growth gradient associated with the stimulus s can be written as

$$\boldsymbol{\delta}^s(s,t) := \frac{\dot{\varepsilon}_v(s,t)}{r}\int_{-\infty}^{t}\mu_s(s,\tau;t)\mathbf{k}_1^s(s,\tau;t)\,\mathrm{d}\tau \tag{S1.38}$$

for an appropriate choice of the response function μ_s.

When applying equation (S1.37) to gravitropism, we have two choices for the stimulus s, which are illustrated in Fig. S2. One possibility is to approximate the stimulus with the vector of gravitational acceleration g, thus neglecting the microscopic description of how gravity is sensed by plant organs. On the contrary, a more accurate choice is to consider the stimulus as perceived by the gravity sensing apparatus. In particular, it is widely accepted that plant organs sense gravity through the sedimentation of starch-filled plastids, called statoliths, into specialized cells, called statocytes, which are found along the shoot growing zone and in the root caps (Chauvet et al., 2019; Nakamura et al., 2019). In this case, we can assume the stimulus s to be given by the average outer normal to the free surface of the pile of statoliths, as shown in Fig. S2C. More specifically, by extending the approach taken by Chauvet et al. (2019) to the three-dimensional case, we can model the statoliths free surface as a plane with normal $\mathbf{h}$, whose dynamics

is a viscous relaxation to $-\mathbf{g}$. As shown in Section S2, the time evolution of $\mathbf{h}$ is governed by

$$\sum_j \dot{h}_j \mathbf{d}_j = \frac{1}{\tau_a} \mathbf{h} \times (\mathbf{h} \times \mathbf{g}) \,, \tag{S1.39}$$

where τ_a is the characteristic time for the statoliths avalanche dynamics and $h_j := \mathbf{h} \cdot \mathbf{d}_j$. As elsewhere, a superimposed dot denotes the material time derivative.

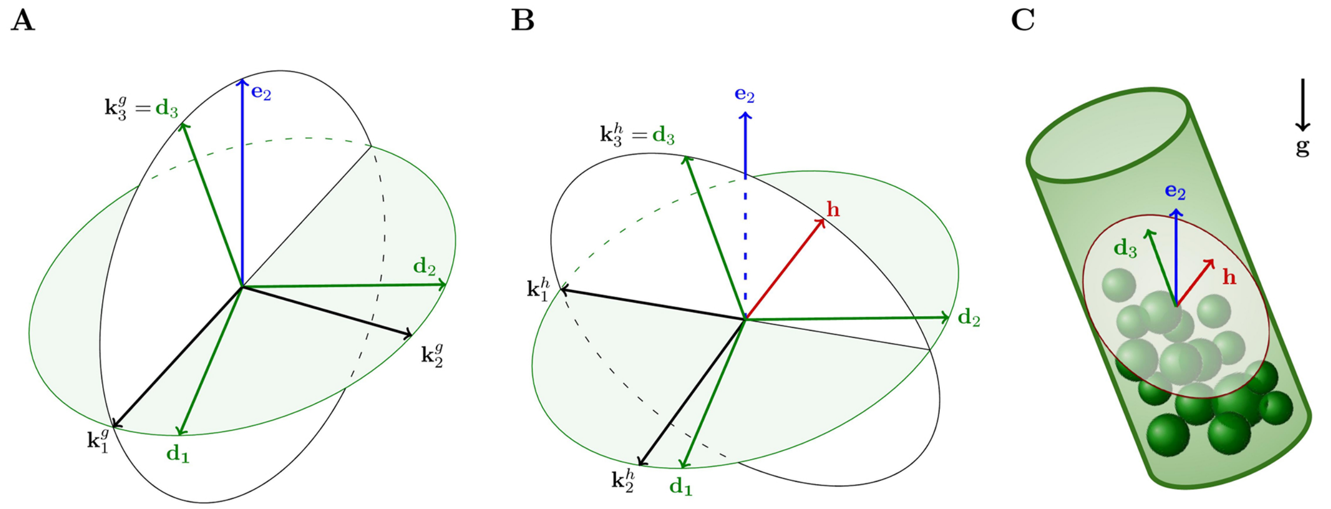

Figure S2. **(A-B)** Illustration of the orthonormal bases exploited to define the gravitropic responses. **(A)** The basis $\{\mathbf{k}_1^g, \mathbf{k}_2^g, \mathbf{k}_3^g\}$ is constructed by defining $\mathbf{k}_1^g$ as the unit vector lying on the stem cross section and having the most negative $\mathbf{e}_2$-component, and setting $\mathbf{k}_3^g := \mathbf{d}_3$ and $\mathbf{k}_2^g := \mathbf{k}_3^g \times \mathbf{k}_1^g$. **(B)** The basis $\{\mathbf{k}_1^h, \mathbf{k}_2^h, \mathbf{k}_3^h\}$ is constructed in a similar manner by defining the unit vector lying on the stem cross section having the most negative $\mathbf{h}$-component, $\mathbf{k}_1^h$, and considering $\mathbf{k}_3^h := \mathbf{d}_3$ and $\mathbf{k}_2^h := \mathbf{k}_3^h \times \mathbf{k}_1^h$. **(C)** Sketch of a single statocyte cell where $\mathbf{h}$ is the average outer normal to the free surface of the pile of statoliths.

S1.4.3 Proprioception

A number of experimental studies have pointed out the existence of an independent straightening mechanism, often referred to as proprioception, autotropism or autostraightening, which is triggered by bending of the organ (Okamoto et al., 2015). Following Bastien et al. (2014) and Bastien and Meroz (2016), we assume that such a straightening response is driven by the geometric curvature of the organ, *i.e.*, $\kappa = \sigma^{-1}(u_1^2 + u_2^2)^{1/2}$, thus producing a growth gradient parallel to the visible normal vector $\boldsymbol{\nu} := \kappa^{-1}\partial_s \mathbf{t}$, where $\mathbf{t} = \partial_s \mathbf{p}$ is the tangent to the rod axis. In other terms, we prescribe

$$\boldsymbol{\delta}^p(s,t) := \dot{\varepsilon}_v(s,t) \int_{-\infty}^{t} \mu_p(s,\tau;t) \boldsymbol{\nu} \, \mathrm{d}\tau \tag{S1.40}$$

for an appropriate response function μ_p that is proportional to κ.

S2 THE STATOLITHS AVALANCHE DYNAMICS

If compared to the purely phenomenological model of Sachs' sine law (von Sachs, 1882), plant gravitropic responses can be refined by including the dynamics of the statoliths avalanche in plant cells. This has been demonstrated to be the microscopic mechanism through which plant shoots and roots perceive the direction of gravity (Nakamura et al., 2019; Chauvet et al., 2019). We assume the free surface of the statoliths pile in a statocyte cell to be planar and the normal given by the unit vector $\mathbf{h}(s,t)$, as depicted in Fig. S3. Since our reasoning holds for any fixed cross section coordinate s, in the following we omit the dependence on such a parameter.

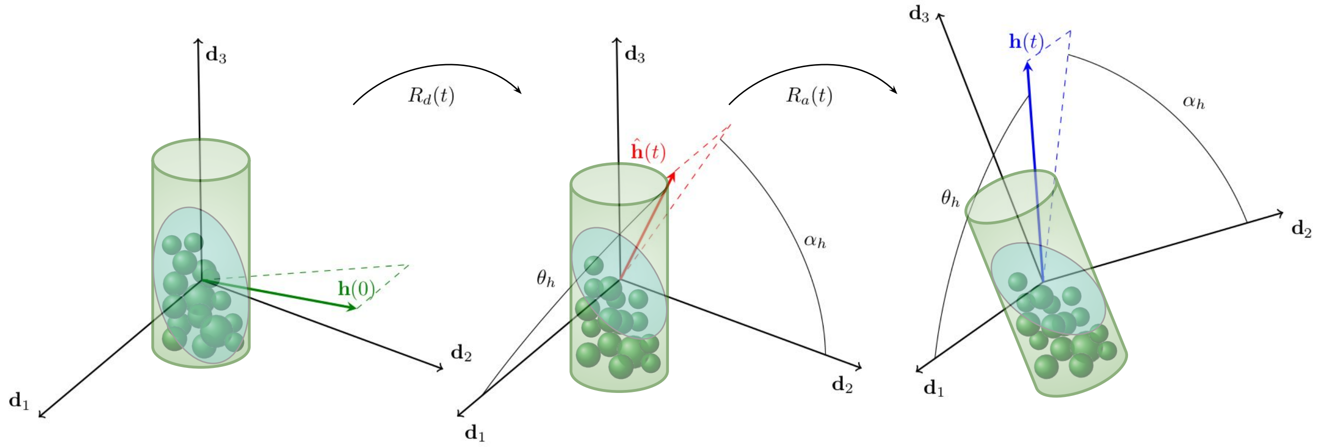

Figure S3. Statoliths avalanche dynamics in a statocyte. The motion of the free surface of piled statoliths can be decomposed into two rotations as in (S2.1): $\hat{\mathbf{h}}(t) = \mathbf{R}_a(t)\mathbf{h}(0)$ is the orientation as described by an observer co-moving with the directors and $\mathbf{h}(t) = \mathbf{R}_d(t)\hat{\mathbf{h}}(t)$ is the orientation as seen by an external observer. Here the unit vector $\mathbf{h}$ is parameterized by two angles defined with respect to the directors: θ_h is the angle between $\mathbf{h}$ and $\mathbf{d}_1$ and α_h is the angle between $(\mathbf{I} - \mathbf{d}_1 \otimes \mathbf{d}_1)\mathbf{h}$ and $\mathbf{d}_2$.

Fig. S3 shows the decomposition of the motion of $\mathbf{h}$ into two dynamics, namely,

$$\mathbf{h}(t) = \mathbf{R}_d(t)\mathbf{R}_a(t)\mathbf{h}(0), \tag{S2.1}$$

where, for any t, $\mathbf{R}_a(t)$ and $\mathbf{R}_d(t)$ are rotations. Here, $\mathbf{R}_a(t)$ can be thought of as the viscous relaxation of the statoliths pile relative to the statocyte so that $\hat{\mathbf{h}}(t) := \mathbf{R}_a(t)\mathbf{h}(0)$ is the dynamics as described by an observer co-moving with the directors, while $\mathbf{R}_d(t)$ is the rotation of the material frame, that is, of the statocyte itself. We prescribe the dynamics of $\hat{\mathbf{h}}$ as a viscous relaxation towards $\mathbf{R}_d(t)^T\mathbf{e}_2$, *i.e.*,

$$\dot{\hat{\mathbf{h}}}(t) = -\frac{1}{\tau_a}\left(\mathbf{R}_d(t)^T\mathbf{e}_2 \times \hat{\mathbf{h}}(t)\right) \times \hat{\mathbf{h}}(t), \tag{S2.2}$$

where τ_a is the characteristic time scale for the statoliths avalanche dynamics. By taking the time derivative of equation (S2.1) and making use of equation (S2.2), we arrive at

$$\begin{aligned}\dot{\mathbf{h}}(t) &= \left(\dot{\mathbf{R}}_d(t)\mathbf{R}_a(t) + \mathbf{R}_d(t)\dot{\mathbf{R}}_a(t)\right)\mathbf{h}(0)\\ &= \dot{\mathbf{R}}_d(t)\mathbf{R}_d(t)^T\mathbf{h}(t) + \mathbf{R}_d(t)\dot{\mathbf{R}}_a(t)\mathbf{R}_a(t)^T\hat{\mathbf{h}}(t)\end{aligned}$$

$$= \mathbf{w}(t) \times \mathbf{h}(t) + \mathbf{R}_d(t) \left[-\frac{1}{\tau_a} \left(\mathbf{R}_d(t)^T \mathbf{e}_2 \times \hat{\mathbf{h}}(t) \right) \times \mathbf{R}_d(t)^T \mathbf{h}(t) \right]$$

$$= \left(\mathbf{w}(t) + \frac{1}{\tau_a} \mathbf{h}(t) \times \mathbf{e}_2 \right) \times \mathbf{h}(t), \tag{S2.3}$$

where $\mathbf{w}(t)$ is the spin, namely, the axial vector associated with $\mathbf{R}_d(t)$. In other terms,

$$\dot{\mathbf{h}}(t) - \mathbf{w}(t) \times \mathbf{h}(t) = \frac{1}{\tau_a} (\mathbf{h}(t) \times \mathbf{e}_2) \times \mathbf{h}(t), \tag{S2.4}$$

and, by using the kinematic relationship $\dot{\mathbf{d}}_j = \mathbf{w} \times \mathbf{d}_j$, we get

$$\sum_j \dot{h}_j(t) \mathbf{d}_j(t) = \frac{1}{\tau_a} (\mathbf{h}(t) \times \mathbf{e}_2) \times \mathbf{h}(t), \tag{S2.5}$$

where $h_j := \mathbf{h} \cdot \mathbf{d}_j$. In terms of components, equation (S2.5) reads

$$\dot{h}_j(t) = \frac{1}{\tau_a} \left[d_{j2}(t) - (\mathbf{h}(t) \cdot \mathbf{e}_2)(\mathbf{h}(t) \cdot \mathbf{d}_j(t)) \right]$$

$$= \frac{1}{\tau_a} \left[d_{j2}(t) - h_j(t) \sum_i h_i(t) d_{i2}(t) \right] \qquad \forall j, \tag{S2.6}$$

where $d_{ij} := \mathbf{d}_i \cdot \mathbf{e}_j$ for all $i, j = 1, 2, 3$. However, we notice that the components of $\mathbf{h}$ are not independent one from the other, due to the constraint on the norm. *i.e.*, $||\mathbf{h}|| = 1$. Then, from the practical point of view, it is convenient to parameterize $\mathbf{h}$ with two angles defined with respect to a certain frame of reference. One possibility is to consider the angles that $\mathbf{h}$ forms with $\mathbf{d}_1$ and $\mathbf{d}_2$, as shown in Fig. S3. In this case, θ_h is the angle between $\mathbf{h}$ and $\mathbf{d}_1$, while α_h is the angle between $(\mathbf{I} - \mathbf{d}_1 \otimes \mathbf{d}_1)\mathbf{h}$ and $\mathbf{d}_2$, so that

$$\mathbf{h} = \cos\theta_h \mathbf{d}_1 + \sin\theta_h \cos\alpha_h \mathbf{d}_2 + \sin\theta_h \sin\alpha_h \mathbf{d}_3. \tag{S2.7}$$

Then

$$\dot{h}_1 = -\dot{\theta}_h \sin\theta_h, \tag{S2.8a}$$

$$\dot{h}_2 = \dot{\theta}_h \cos\theta_h \cos\alpha_h - \dot{\alpha}_h \sin\theta_h \sin\alpha_h, \tag{S2.8b}$$

$$\dot{h}_3 = \dot{\theta}_h \cos\theta_h \sin\alpha_h + \dot{\alpha}_h \sin\theta_h \cos\alpha_h, \tag{S2.8c}$$

whence

$$\cos\theta_h \left(\cos\alpha_h \dot{h}_2 + \sin\alpha_h \dot{h}_3 \right) - \sin\theta_h \dot{h}_1 = \dot{\theta}_h, \tag{S2.9a}$$

$$\cos\alpha_h \dot{h}_3 - \sin\alpha_h \dot{h}_2 = \dot{\alpha}_h \sin\theta_h. \tag{S2.9b}$$

Therefore, for $\sin\theta_h \neq 0$, equations (S2.9) provide the evolution laws (S2.5) in terms of the angles θ_h and α_h.

S3 STABILITY ANALYSES

In this section we report the stability analyses of the model discussed in Section 3 of the main text. We start by summarizing the governing equations for the model of growing plant shoots. Then, upon introducing their representation in terms of Euler angles, we proceed to study the reduced model described in Section 2.2 of the main text, by gradually exploring the effects of gravitropic and proprioceptive responses.

S3.1 Summary of the model for growing plant shoots

In Section 2.1 of the main text we introduced a rod model for the study of growing plant shoots, whose governing equations read

$$\frac{\partial s}{\partial S}(S,t) = \lambda(S,t), \tag{S3.1a}$$

$$\frac{1}{\lambda(S,t)}\frac{\partial \lambda}{\partial t}(S,t) = \begin{cases} 0 & \text{if } s(S,t) \leq \ell(t) - \ell_g, \\ \frac{1}{\tau_g} & \text{if } s(S,t) > \ell(t) - \ell_g, \end{cases} \tag{S3.1b}$$

$$\mathbf{m}'(s,t) = -\, q\,(\ell(t) - s)\,\mathbf{e}_2 \times \mathbf{d}_3(s,t), \tag{S3.1c}$$

$$\mathbf{m} = \sum_j K_j \left(u_j - u_j^\star\right) \mathbf{d}_j, \tag{S3.1d}$$

$$E(s,t) = E_1 - (E_1 - E_0)\, e^{-\frac{1}{\tau_\ell}\max\{0, t - t^\star(S(s,t))\}}, \tag{S3.1e}$$

$$\sum_j \dot{h}_j(s,t)\mathbf{d}_j(s,t) = \frac{1}{\tau_a}(\mathbf{h}(s,t) \times \mathbf{e}_2) \times \mathbf{h}(s,t), \tag{S3.1f}$$

$$\begin{aligned}\dot{u}_1^\star(s,t) = {} & \alpha\frac{\dot{\varepsilon}(s,t)}{r}\cos(2\pi t/\tau_e) - \beta\frac{\dot{\varepsilon}(s,t)}{r\tau_m}\int_{-\infty}^{t-\tau_r} e^{-\frac{1}{\tau_m}(t-\tau_r-\tau)} h_2(s,\tau)\,\mathrm{d}\tau \\ & - \eta\frac{\dot{\varepsilon}(s,t)}{\bar{\tau}_m}\int_{-\infty}^{t-\bar{\tau}_r} e^{-\frac{1}{\bar{\tau}_m}(t-\bar{\tau}_r-\tau)} u_1(s,\tau)\,\mathrm{d}\tau,\end{aligned} \tag{S3.1g}$$

$$\begin{aligned}\dot{u}_2^\star(s,t) = {} & \alpha\frac{\dot{\varepsilon}(s,t)}{r}\sin(2\pi t/\tau_e) + \beta\frac{\dot{\varepsilon}(s,t)}{r\tau_m}\int_{-\infty}^{t-\tau_r} e^{-\frac{1}{\tau_m}(t-\tau_r-\tau)} h_1(s,\tau)\,\mathrm{d}\tau \\ & - \eta\frac{\dot{\varepsilon}(s,t)}{\bar{\tau}_m}\int_{-\infty}^{t-\bar{\tau}_r} e^{-\frac{1}{\bar{\tau}_m}(t-\bar{\tau}_r-\tau)} u_2(s,\tau)\,\mathrm{d}\tau,\end{aligned} \tag{S3.1h}$$

where all variables and parameters are defined as in the main text, primes denote differentiation with respect to the parameter s and dots denote material time derivatives. We recall that equations (S3.1a)-(S3.1b) define the subapical growth law, equation (S3.1c) follows from the balance of linear and angular momentum where $\mathbf{m}$ is the resultant contact couple given by the constitutive law of (S3.1d). Equation (S3.1e) is the lignification law, equation (S3.1f) governs the statoliths avalanche dynamics, and equations (S3.1g)-(S3.1h) are the evolution laws for the spontaneous strains.

S3.2 Representation in terms of Euler angles

The nine components of the directors $\{\mathbf{d}_j\}$ are not independent, due to the orthonormality constraints. Then it is possible to represent the directors in terms of three independent angles, the Euler angles, so that the orthonormality constraints are automatically fulfilled. Although this representation introduces a polar singularity leading to an ambiguity of the representation, this can be successfully adopted in our setting, upon a careful choice of the notation for the Euler angles.

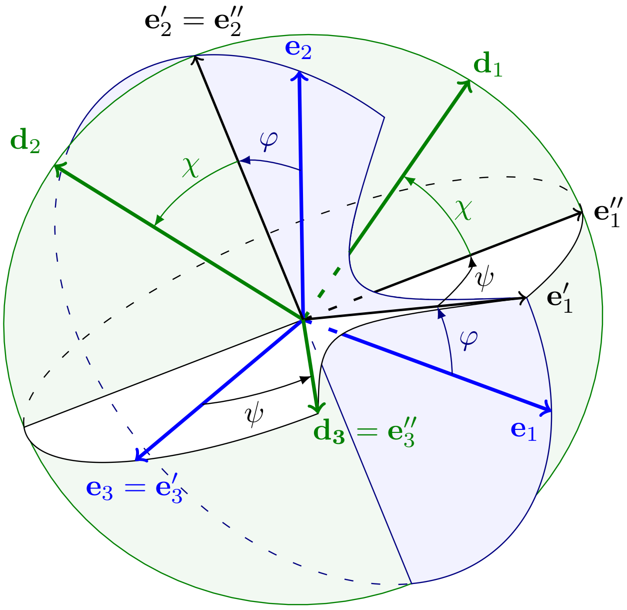

Figure S4. The relationship of the directors $\{\mathbf{d}_j\}$ to the fixed basis $\{\mathbf{e}_j\}$ via the Euler angles χ, ψ and φ.

We describe the rotation mapping the fixed basis $\{\mathbf{e}_j\}$ to the basis of directors $\{\mathbf{d}_j\}$ by means of the following three successive rotations:

1. A rotation by an angle φ about the $\mathbf{e}_3$-axis;
2. A rotation by an angle ψ about the rotated $\mathbf{e}_2$-axis, denoted by $\mathbf{e}_2'$;
3. A rotation by an angle χ about the rotated $\mathbf{e}_3$-axis, denoted by $\mathbf{e}_3''$.

Such a decomposition is illustrated in Fig. S4 and it is well defined if $\psi \neq 0$, otherwise the Euler angles are not uniquely determined by the directors, since only the sum $\chi + \varphi$ can be established. On the other hand, the directors are always uniquely determined by the three angles as

$$\mathbf{d}_1 = (\cos\chi\cos\psi\cos\varphi - \sin\chi\sin\varphi)\,\mathbf{e}_1 + (\cos\chi\cos\psi\sin\varphi + \sin\chi\cos\varphi)\,\mathbf{e}_2 - \cos\chi\sin\psi\,\mathbf{e}_3, \tag{S3.2a}$$

$$\mathbf{d}_2 = -\,(\sin\chi\cos\psi\cos\varphi + \cos\chi\sin\varphi)\,\mathbf{e}_1 - (\sin\chi\cos\psi\sin\varphi - \cos\chi\cos\varphi)\,\mathbf{e}_2 + \sin\chi\sin\psi\,\mathbf{e}_3, \tag{S3.2b}$$

$$\mathbf{d}_3 = \sin\psi\cos\varphi\,\mathbf{e}_1 + \sin\psi\sin\varphi\,\mathbf{e}_2 + \cos\psi\,\mathbf{e}_3. \tag{S3.2c}$$

Consequently, the strains can be written as

$$u_1 = \frac{\partial\psi}{\partial s}\sin\chi - \frac{\partial\varphi}{\partial s}\cos\chi\sin\psi, \quad u_2 = \frac{\partial\psi}{\partial s}\cos\chi + \frac{\partial\varphi}{\partial s}\sin\chi\sin\psi \quad \text{and} \quad u_3 = \frac{\partial\chi}{\partial s} + \frac{\partial\varphi}{\partial s}\cos\psi. \tag{S3.3}$$

Moreover, denoting by m_j the components of the resultant moment with respect to the fixed basis $\{\mathbf{e}_j\}$, the constitutive assumption (9) in the main text leads to

$$\begin{aligned} m_1 = & EI\,\{\cos\psi\cos\varphi\,[(u_1 - u_1^\star)\cos\chi - (u_2 - u_2^\star)\sin\chi] \\ & - \sin\varphi\,[(u_1 - u_1^\star)\sin\chi + (u_2 - u_2^\star)\cos\chi]\} + \mu J\,(u_3 - u_3^\star)\sin\psi\cos\varphi, \end{aligned} \tag{S3.4a}$$

$$m_2 = EI\,\{\cos\psi\sin\varphi\,[(u_1 - u_1^\star)\cos\chi - (u_2 - u_2^\star)\sin\chi] + \cos\varphi\,[(u_1 - u_1^\star)\sin\chi + (u_2 - u_2^\star)\cos\chi]\} + \mu J\,(u_3 - u_3^\star)\sin\psi\sin\varphi, \tag{S3.4b}$$

$$m_3 = -\,EI\sin\psi\,[(u_1 - u_1^\star)\cos\chi - (u_2 - u_2^\star)\sin\chi] + \mu J\,(u_3 - u_3^\star)\cos\psi. \tag{S3.4c}$$

S3.3 Analysis of the reduced model for short times

Under additional hypotheses on the times scales stated in Section 2.2 of the main text, we reduce (S3.1) to get a model suitable for a theoretical study on circumnutations. By focusing on short time periods compared to τ_g and τ_ℓ, we assume a shoot of constant length ℓ with constant Young's modulus E, whose evolution is governed by

$$\mathbf{m}'(s,t) = -\,q\,(\ell - s)\,\mathbf{e}_2 \times \mathbf{d}_3(s,t), \tag{S3.5a}$$

$$\dot{u}_1^\star(s,t) = \frac{\alpha}{r\tau_g}\cos\left(\frac{2\pi t}{\tau_e}\right) - \frac{\beta}{r\tau_m\tau_g}\int_{-\infty}^{t-\tau_r} e^{-\frac{1}{\tau_m}(t-\tau_r-\tau)} d_{22}(s,\tau)\,\mathrm{d}\tau - \frac{\eta}{\bar{\tau}_m\tau_g}\int_{-\infty}^{t-\bar{\tau}_r} e^{-\frac{1}{\bar{\tau}_m}(t-\bar{\tau}_r-\tau)} u_1(s,\tau)\,\mathrm{d}\tau, \tag{S3.5b}$$

$$\dot{u}_2^\star(s,t) = \frac{\alpha}{r\tau_g}\sin\left(\frac{2\pi t}{\tau_e}\right) + \frac{\beta}{r\tau_m\tau_g}\int_{-\infty}^{t-\tau_r} e^{-\frac{1}{\tau_m}(t-\tau_r-\tau)} d_{12}(s,\tau)\,\mathrm{d}\tau - \frac{\eta}{\bar{\tau}_m\tau_g}\int_{-\infty}^{t-\bar{\tau}_r} e^{-\frac{1}{\bar{\tau}_m}(t-\bar{\tau}_r-\tau)} u_2(s,\tau)\,\mathrm{d}\tau, \tag{S3.5c}$$

where $d_{ij} := \mathbf{d}_i \cdot \mathbf{e}_j$. Then, in terms of the Euler angles introduced in Section S3.2, we get

$$m_1'(s,t) = -\,q\,(\ell - s)\cos\psi(s,t), \tag{S3.6a}$$

$$m_2'(s,t) = 0, \tag{S3.6b}$$

$$m_3'(s,t) = q\,(\ell - s)\sin\psi(s,t)\cos\varphi(s,t), \tag{S3.6c}$$

$$\dot{u}_1^\star(s,t) = \frac{\alpha}{r\tau_g}\cos\left(\frac{2\pi t}{\tau_e}\right) + \frac{\beta}{r\tau_m\tau_g}\int_{-\infty}^{t-\tau_r} e^{-\frac{1}{\tau_m}(t-\tau_r-\tau)}\,(\sin\chi\cos\psi\sin\varphi - \cos\chi\cos\varphi)\big|_{(s,\tau)}\,\mathrm{d}\tau - \frac{\eta}{\bar{\tau}_m\tau_g}\int_{-\infty}^{t-\bar{\tau}_r} e^{-\frac{1}{\bar{\tau}_m}(t-\bar{\tau}_r-\tau)}\,\left[\psi'\sin\chi - \varphi'\cos\chi\sin\psi\right]\big|_{(s,\tau)}\,\mathrm{d}\tau, \tag{S3.6d}$$

$$\dot{u}_2^\star(s,t) = \frac{\alpha}{r\tau_g}\sin\left(\frac{2\pi t}{\tau_e}\right) + \frac{\beta}{r\tau_m\tau_g}\int_{-\infty}^{t-\tau_r} e^{-\frac{1}{\tau_m}(t-\tau_r-\tau)}\,(\cos\chi\cos\psi\sin\varphi + \sin\chi\cos\varphi)\big|_{(s,\tau)}\,\mathrm{d}\tau - \frac{\eta}{\bar{\tau}_m\tau_g}\int_{-\infty}^{t-\bar{\tau}_r} e^{-\frac{1}{\bar{\tau}_m}(t-\bar{\tau}_r-\tau)}\,\left[\psi'\cos\chi + \varphi'\sin\chi\sin\psi\right]\big|_{(s,\tau)}\,\mathrm{d}\tau, \tag{S3.6e}$$

to be solved for appropriate boundary conditions and initial data.

In the following we explore the contribution of gravitropic and proprioceptive responses.

S3.3.1 Graviceptive model: $\boldsymbol{\alpha = \eta = 0}$ and $\boldsymbol{\beta > 0}$

Let us consider the rod model (S3.6) for $\alpha = \eta = 0$. We notice that the planar case studied in (Agostinelli et al., 2020) is recovered by confining the rod to the $(\mathrm{e}_1, \mathrm{e}_2)$-plane (*i.e.*, $\psi(s,t) = \pi/2$ and $\chi(s,t) = 0$ for all s and t), and writing the governing equations in terms of the angle $\theta := \pi/2 - \varphi$. In this case the model

suffers an instability and exhibits the onset of a limit cycle as the shoot's length exceeds a critical value. Guided by this result, we extend the analysis to the three-dimensional case.

The steady state solution to problem (S3.6) is given by

$$\chi \equiv 0, \quad \varphi \equiv \frac{\pi}{2}, \quad \psi \equiv \frac{\pi}{2}, \quad u_1^\star \equiv 0, \quad u_2^\star \equiv 0, \tag{S3.7}$$

which corresponds to the straight position along $\mathbf{e}_2$, the axis of gravity. By assuming sufficient regularity, we take the time derivative of equations (S3.6d) and (S3.6e), and we linearize the problem about the equilibrium solution (S3.7), arriving at

$$\begin{aligned}
EI\left(\psi'(s,t) - u_2^\star(s,t)\right)' &= -\,q\,(\ell - s)\left(\psi(s,t) - \frac{\pi}{2}\right), && \text{(S3.8a)}\\
\chi''(s,t) &= 0, && \text{(S3.8b)}\\
EI\left(\varphi'(s,t) + u_1^\star(s,t)\right)' &= -\,q\,(\ell - s)\,,\left(\varphi(s,t) - \frac{\pi}{2}\right), && \text{(S3.8c)}\\
\ddot{u}_1^\star(s,t) &= -\,\frac{1}{\tau_m}\dot{u}_1^\star(s,t) + \frac{\beta}{r\tau_g\tau_m}\left(\varphi(s,t-\tau_r) - \frac{\pi}{2}\right), && \text{(S3.8d)}\\
\ddot{u}_2^\star(s,t) &= -\,\frac{1}{\tau_m}\dot{u}_2^\star(s,t) - \frac{\beta}{r\tau_g\tau_m}\left(\psi(s,t-\tau_r) - \frac{\pi}{2}\right). && \text{(S3.8e)}
\end{aligned}$$

This system of equations is supplemented by the following linearized boundary and initial conditions,

$$\begin{aligned}
\psi(0,t) &= \frac{\pi}{2}, \qquad \psi'(\ell,t) - u_2^\star(\ell,t) = 0, && \text{(S3.9a)}\\
\chi(0,t) &= 0, \qquad \chi'(\ell,t) = 0, && \text{(S3.9b)}\\
\varphi(0,t) &= \frac{\pi}{2}, \qquad \varphi'(\ell,t) + u_1^\star(\ell,t) = 0, && \text{(S3.9c)}
\end{aligned}$$

holding $\forall\, t > 0$ as the basal end is clamped and the apical end is torque free, and

$$\begin{aligned}
\varphi(s,t) &= \varphi_0(s,t), \qquad \psi(s,t) = \psi_0(s,t), && \text{(S3.10a)}\\
u_1^\star(s,0) &= u_{1,0}^\star(s), \qquad u_2^\star(s,0) = u_{2,0}^\star(s), && \text{(S3.10b)}
\end{aligned}$$

prescribing respectively the past history of the angular coordinates and the initial datum for the evolution of the spontaneous strains for all $s \in [0, \ell]$.

As for the angle χ, equations (S3.8b) and (S3.9b) yield $\chi(s,t) = 0$ for all s and t. Moreover, by assuming sufficient regularity, we can combine the time-derivatives of (S3.8a) and (S3.8c) with the space-derivatives of (S3.8e) and (S3.8d) respectively, so that we get

$$\ddot{\psi}''(s,t) + \frac{1}{\tau_m}\dot{\psi}''(s,t) + \frac{q(\ell - s)}{K_1}\left(\ddot{\psi}(s,t) + \frac{1}{\tau_m}\dot{\psi}(s,t)\right) + \frac{\beta}{r\tau_m\tau_g}\psi'(s,t-\tau_r) = 0, \tag{S3.11a}$$

$$\ddot{\varphi}''(s,t) + \frac{1}{\tau_m}\dot{\varphi}''(s,t) + \frac{q(\ell - s)}{K_1}\left(\ddot{\varphi}(s,t) + \frac{1}{\tau_m}\dot{\varphi}(s,t)\right) + \frac{\beta}{r\tau_m\tau_g}\varphi'(s,t-\tau_r) = 0, \tag{S3.11b}$$

along with the boundary conditions (S3.9a)$_1$, (S3.9c)$_1$ and

$$\ddot{\psi}'(\ell,t)+\frac{1}{\tau_m}\dot{\psi}'(\ell,t)+\frac{\beta}{r\tau_m\tau_g}\left(\psi(\ell,t-\tau_r)-\frac{\pi}{2}\right)=0, \tag{S3.12a}$$

$$\ddot{\varphi}'(\ell,t)+\frac{1}{\tau_m}\dot{\varphi}'(\ell,t)+\frac{\beta}{r\tau_m\tau_g}\left(\varphi(\ell,t-\tau_r)-\frac{\pi}{2}\right)=0, \tag{S3.12b}$$

holding $\forall\, t>0$ and resulting from time differentiation of (S3.9a)$_2$ and (S3.9c)$_2$.

We notice that equations (S3.11) are decoupled and, up to a shift by $\pi/2$, they are equivalent to the linearization of the planar model about the equilibrium of $\theta\equiv 0$ (Agostinelli et al., 2020). Therefore we can rely on the analysis carried out for the planar model to conclude that the trivial equilibrium becomes unstable when the same critical length is attained.

S3.3.2 Microgravity: $\boldsymbol{\alpha}=\boldsymbol{\beta}=\mathbf{0}$, $\boldsymbol{\eta}>\mathbf{0}$, and $\boldsymbol{q}=\mathbf{0}$

For $\beta=0$ and $q=0$, virtual and current configuration coincide (*i.e.*, $u_j^\star=u_j$ for all j), so that equations (S3.5) reduce to

$$\dot{u}_j(s,t)=-\frac{\eta}{\bar{\tau}_m\tau_g}\int_{-\infty}^{t-\bar{\tau}_r}e^{-\frac{1}{\bar{\tau}_m}(t-\bar{\tau}_r-\tau)}u_j(s,\tau)\,\mathrm{d}\tau, \tag{S3.13}$$

for $j=1,2$. By assuming sufficient regularity, a time differentiation yields

$$\ddot{u}_j(s,t)+\frac{1}{\bar{\tau}_m}\dot{u}_j(s,t)+\frac{\eta}{r\tau_g\bar{\tau}_m}u_j(s,t-\bar{\tau}_r)=0, \tag{S3.14}$$

for $j=1,2$, which can be restated in dimensionless form as

$$\ddot{\hat{u}}_j(\hat{s},\hat{t})+\frac{\bar{\tau}_r}{\bar{\tau}_m}\dot{\hat{u}}_j(\hat{s},\hat{t})+\frac{\eta\,\bar{\tau}_r^2}{\bar{\tau}_m\tau_g}\hat{u}_j(\hat{s},\hat{t}-1)=0, \tag{S3.15}$$

where $\hat{u}_j(\hat{s},\hat{t}):=u_j(\hat{s}\ell,\hat{t}\tau_r)$ for $j=1,2$, and dots and primes denote differentiation with respect to $\hat{t}:=t/\bar{\tau}_r$ and $\hat{s}:=s/\ell$, respectively.

Since equation (S3.15) does not contain space derivatives, we can rely on the theory of retarded functional differential equations (RFDEs) by considering the space variable as a parameter. Indeed, given the problem

$$\ddot{u}(s,t)+a\dot{u}(s,t)+bu(s,t-1)=0,\qquad s\in[0,\ell_0]\,,\ t>1, \tag{S3.16a}$$

$$u(s,t)=u_0(s,t),\qquad s\in[0,\ell_0]\,,\ t\in[0,1]\,, \tag{S3.16b}$$

with $a>0$ and an initial datum u_0 that is regular enough, say $u_0\in C^\infty$, we can consider the solution $u(s,t):=u_s(t)$ where $u_s(t)$ is the unique solution to

$$\ddot{v}(t)+a\dot{v}(t)+bv(t-1)=0,\qquad t>1, \tag{S3.17a}$$

$$v(t)=u_0(s,t),\qquad t\in[0,1]\,, \tag{S3.17b}$$

for any fixed $s\in[0,\ell_0]$. Then the regularity of $u(s,t)$ with respect to s follows from the results on the continuous dependence of solutions to RFDEs on initial data (Hale and Lunel, 1993). Moreover, we can

exploit the stability analysis of the trivial equilibrium of (S3.17) to learn something about the solution $u(s,t)$. To this aim, we restate the following lemma that holds for the characteristic equation associated with (S3.17) (Agostinelli et al., 2020; Somolinos, 1978).

LEMMA S3.1. *Consider the equation*

$$\left(\omega^2 + a\omega\right) e^{\omega} + b = 0, \tag{S3.18}$$

for $b > 0$, and let ξ_b be the unique solution of $\xi^2 = b\cos(\xi)$ in $(0, \pi/2)$ and $a_b := \sin(\xi_b)b/\xi_b$. Then the following holds for equation (S3.18)*:*

1. *All roots have negative real parts if and only if $a > a_b$;*
2. *For $a = a_b$, $\pm i\xi_b$ is the only pair of simple imaginary roots. In particular, no other root is an integer multiple of $i\xi_b$;*
3. *There exists an $\epsilon > 0$ and a root $\omega(a)$ that is continuously differentiable in $(a_b - \epsilon, a_b + \epsilon)$ s.t. $\omega(a_b) = i\xi_b$ and $\mathrm{Re}\left(\omega'(a_b)\right) < 0$;*
4. *For each $a < a_b$, there exist precisely two roots ω with $\mathrm{Re}\left(\omega\right) > 0$ and $\mathrm{Im}\left(\omega\right) \in (-\pi, \pi)$.*

In addition to this, we can show the following.

LEMMA S3.2. *Consider equation* (S3.18) *for $b > 0$ and let $b_a := (a + 2\tilde{\omega})\, e^{\tilde{\omega}}$ where $\tilde{\omega} := (\sqrt{4 + a^2} - a - 2)/2$. Then for $b < b_a$ there exist precisely two real roots, which coincide for $b = b_a$, whereas there exist no real roots for $b > b_a$.*

PROOF. Let us define $y(\omega) := \omega^2 + a\omega$ and $z(\omega) := -be^{-\omega}$. By means of the graphical method, one can show that there are at most two real intersections between the graphs of y and z. If there is a single distinct real root $\tilde{\omega}$, then it is such that

$$y(\tilde{\omega}) = z(\tilde{\omega}) \quad \text{and} \quad \frac{\partial y}{\partial \omega}(\tilde{\omega}) = \frac{\partial z}{\partial \omega}(\tilde{\omega}), \tag{S3.19}$$

namely, $\tilde{\omega}^2 + a\tilde{\omega} + be^{-\tilde{\omega}} = 0$ and $2\tilde{\omega} + a = be^{-\tilde{\omega}}$. Therefore $\tilde{\omega}$ needs to solve $\tilde{\omega}^2 + (2 + a)\tilde{\omega} + a = 0$, whose solutions are

$$\omega_{\pm} = \frac{-2 - a \pm \sqrt{4 + a^2}}{2}. \tag{S3.20}$$

Since $2\tilde{\omega} + a = be^{-\tilde{\omega}} > 0$, we conclude that the only admissible root is given by $\tilde{\omega} = \omega_+$ and that $b = (a + 2\tilde{\omega})e^{\tilde{\omega}} =: b_a$.

Finally we can prove the following facts.

THEOREM S3.3. *Consider problem* (S3.16) *and the solution $u(s,t) := u_s(t)$ where u_s solves* (S3.17) *for any $s \in [0, \ell_0]$. Moreover, let a_b be defined as in Lemma S3.1. Then,*

1. *if $a > a_b$, the trivial equilibrium of* (S3.16a) *is stable and there exists $\delta > 0$ such that $||u_0||_\infty < \delta$ implies that, for any fixed $s \in [0, \ell_0]$, $|u(s,t)| \to 0$ as $t \to \infty$;*
2. *if $a < a_b$, the trivial equilibrium of* (S3.16a) *is unstable.*

Moreover, for $a = a_b$, equation (S3.16a) *admits nontrivial periodic solutions.*

PROOF. Assume that $a > a_b$. Then, by means of Lemma S3.1, for any $\epsilon > 0$, there exists $\delta_\epsilon > 0$ such that $\sup |u_0(s,t)| < \delta_\epsilon$ implies $|u_s(t)| < \epsilon$ for all $t \geq 0$. Moreover, there exists $\delta > 0$ such that $\sup |u_0(s,t)| < \delta$ implies that $|u_s(t)| \to 0$ as $t \to \infty$. It follows that if we take $u_0(s,t)$ such that $\sup |u_0(s,t)| < \min\{\delta_\epsilon, \delta\}$, then $|u_s(t)| < \epsilon$ for all $(s,t) \in [0,\ell_0] \times [0,\infty)$ and, for any fixed $s \in [0,\ell_0]$, $|u(s,t)| \to 0$ as $t \to \infty$.

On the contrary, if $a < a_b$, there exists $\epsilon > 0$ such that for any $\delta > 0$, we find an initial datum $\bar{u}(t)$ for which $\sup |\bar{u}(t)| < \delta$ and the corresponding solution of (S3.17a) verifies $|u(t)| > \epsilon$ for some $t \geq 0$. Then the statement follows by observing that $u(s,t) := u(t)$ solves (S3.16) for the space-independent initial datum $u_0(s,t) := \bar{u}(t)$.

Finally, by applying the results above to equation (S3.15), we conclude that it admits nontrivial periodic solutions when

$$\tau_g = \tau_g^\star := \eta \bar{\tau}_r \frac{\sin(\xi^\star)}{\xi^\star}, \tag{S3.21}$$

where $\xi^\star$ is the unique solution of $\xi \tan \xi = \bar{\tau}_r/\bar{\tau}_m$ in $(0, \pi/2)$. Moreover, the characteristic equation associated with (S3.15) has a pair of conjugate complex roots for

$$\tau_g < \tilde{\tau}_g := \frac{\eta \bar{\tau}_r^2 e^{-\omega_0}}{2\omega_0 \bar{\tau}_m + \bar{\tau}_r}, \tag{S3.22}$$

where $\omega_0 := \left(1 + \bar{\tau}_r^2/(4\bar{\tau}_m^2)\right)^{1/2} - (1 + \bar{\tau}_r/(2\bar{\tau}_m))$, and their real part crosses zero at $\tau_g^\star$, as shown in Fig. S5.

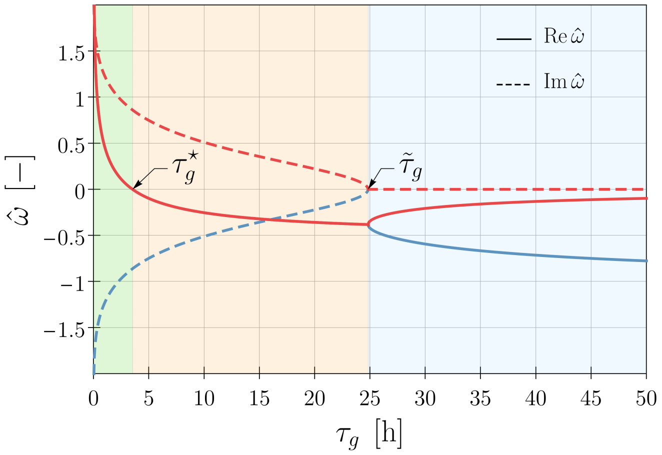

Figure S5. Real (solid lines) and imaginary (dashed lines) part of two roots of the characteristic equation $\hat{\omega}^2 + (\bar{\tau}_r/\bar{\tau}_m)\,\hat{\omega} + [\eta\,\bar{\tau}_r^2/(\bar{\tau}_m\tau_g)]\,e^{-\hat{\omega}} = 0$, as functions of $\tau_g \in [0, 50]$ h and for $\eta = 20$, $\bar{\tau}_r = \bar{\tau}_m = 12$ min, $r = 0.5 \times 10^{-3}$ m. We distinguish three regions corresponding to different dynamical responses: (i) an exponential decay for $\tau_g > \tilde{\tau}_g \approx 24.83$ h (light blue), (ii) a damped oscillation (orange), and (iii) an increasing oscillation for $\tau_g < \tau_g^\star \approx 3.52$ h (green).

Then the trivial straight position is stable for $\tau_g > \tau_g^\star$ and unstable for $\tau_g < \tau_g^\star$. Therefore, when the growth velocity is sufficiently fast, our model exhibits oscillations about the equilibrium configuration also under microgravity conditions, regardless of the shoot length. However, we notice that for the present choice of model parameters, the critical value $\tau_g^\star$ is one order of magnitude smaller than the observed growth times, cf. Table 1 in the main text.

S3.3.3 Proprio-graviceptive model: $\boldsymbol{\alpha} = \mathbf{0}$ and $\boldsymbol{\beta}$, $\boldsymbol{\eta} > \mathbf{0}$

As regards the proprio-graviceptive model corresponding to $\alpha = 0$ and β, $\eta > 0$, we first obtain planar steady-state solutions, which might be useful to estimate the ratio between gravitropic and proprioceptive sensitivities from experimental observations, and then we carry out the stability analysis as done for the graviceptive case.

We first explore the steady-state solutions of the nonlinear model. By confining the rod model (S3.6) to the plane $(\mathbf{e}_1, \mathbf{e}_2)$, and assuming sufficient regularity, we get

$$K_1 \left[\theta'(s,t) - u_1^\star(s,t)\right]' = -\, q(\ell - s)\sin\theta(s,t), \tag{S3.23a}$$

$$r\,\tau_g \dot{u}_1^\star(s,t) = -\,\beta w_g(s,t) - r\,\eta\, w_p(s,t), \tag{S3.23b}$$

$$\tau_m \dot{w}_g(s,t) = -\, w_g(s,t) + \sin\theta(s, t-\tau_r), \tag{S3.23c}$$

$$\bar{\tau}_m \dot{w}_p(s,t) = -\, w_p(s,t) + \theta'(s, t-\tau_r), \tag{S3.23d}$$

as the governing equations, where

$$w_g := \frac{1}{\tau_m}\int_{-\infty}^{t-\tau_r} e^{-\frac{1}{\tau_m}(t-\tau_r-\tau)}\sin\theta(s,\tau)\,\mathrm{d}\tau \quad \text{and} \quad w_p := \frac{1}{\bar{\tau}_m}\int_{-\infty}^{t-\bar{\tau}_r} e^{-\frac{1}{\bar{\tau}_m}(t-\bar{\tau}_r-\tau)}\theta'(s,\tau)\,\mathrm{d}\tau. \tag{S3.24}$$

Then a steady-state solution $\theta(s)$ of (S3.23) needs to solve

$$\hat{\theta}'(\hat{s}) = -\frac{\beta\,\ell}{\eta\, r}\sin\hat{\theta}(\hat{s}), \tag{S3.25}$$

for $\hat{s} \in [0, 1]$, combined with the boundary condition $\hat{\theta}(0) = \theta_0$. Here, $\hat{\theta}(\hat{s}) := \theta(\hat{s}\ell)$ and primes denote differentiation with respect to $\hat{s} := s/\ell$. Then an equilibrium of (S3.23) is given by

$$\hat{\theta}(\hat{s}) = 2\,\mathrm{acot}\left[\cot\left(\tfrac{\theta_0}{2}\right) e^{\frac{\beta}{\eta}\frac{\ell}{r}\hat{s}}\right], \tag{S3.26}$$

for $\hat{s} \in [0, 1]$. Therefore, when converging to (S3.26), the final shape is completely determined by the ratio between the two sensitivities, β/η, while the whole dynamics towards the steady state depends also on the characteristic times, *i.e.*, τ_g, τ_m, τ_r, $\bar{\tau}_m$ and $\bar{\tau}_r$. Since gravitropic and proprioceptive responses generate planar dynamics for initially straight plant shoots, the planar steady-state solution (S3.26) can be used to determine the dimensionless parameter $\beta\ell/(\eta r)$ by fitting the experimental shapes attained in a time period that is short with respect to growth, as already done for the instantaneous version of this model without gravity loads (Bastien et al., 2014).

We proceed by carrying out a stability analysis similar to the graviceptive case. We linearize (S3.6) about the equilibrium (S3.7), thus arriving at

$$\psi''(s,t) = u_2^{\star\prime}(s,t) - \frac{q}{EI}(\ell - s)\left(\psi(s,t) - \frac{\pi}{2}\right), \tag{S3.27a}$$

$$\chi''(s,t) = 0, \tag{S3.27b}$$

$$\varphi''(s,t) = -\, u_1^{\star\prime}(s,t) - \frac{q}{EI}(\ell - s)\left(\varphi(s,t) - \frac{\pi}{2}\right), \tag{S3.27c}$$

$$\dot{u}_1^\star(s,t) = \frac{\beta}{r\tau_m\tau_g}\int_{-\infty}^{t-\tau_r} e^{-\frac{1}{\tau_m}(t-\tau_r-\tau)}\left(\varphi(s,\tau)-\frac{\pi}{2}\right)\mathrm{d}\tau + \frac{\eta}{\bar{\tau}_m\tau_g}\int_{-\infty}^{t-\bar{\tau}_r} e^{-\frac{1}{\bar{\tau}_m}(t-\bar{\tau}_r-\tau)}\varphi'(s,\tau)\,\mathrm{d}\tau, \tag{S3.27d}$$

$$\dot{u}_2^\star(s,t) = -\frac{\beta}{r\tau_m\tau_g}\int_{-\infty}^{t-\tau_r} e^{-\frac{1}{\tau_m}(t-\tau_r-\tau)}\left(\psi(s,\tau)-\frac{\pi}{2}\right)\mathrm{d}\tau - \frac{\eta}{\bar{\tau}_m\tau_g}\int_{-\infty}^{t-\bar{\tau}_r} e^{-\frac{1}{\bar{\tau}_m}(t-\bar{\tau}_r-\tau)}\psi'(s,\tau)\,\mathrm{d}\tau. \tag{S3.27e}$$

By assuming sufficient regularity, we get

$$\begin{aligned}&\dot{\psi}''(s,t) + \frac{\beta}{r\tau_m\tau_g}\int_{-\infty}^{t-\tau_r} e^{-\frac{1}{\tau_m}(t-\tau_r-\tau)}\psi'(s,\tau)\,\mathrm{d}\tau\\ &\quad + \frac{\eta}{\bar{\tau}_m\tau_g}\int_{-\infty}^{t-\bar{\tau}_r} e^{-\frac{1}{\bar{\tau}_m}(t-\bar{\tau}_r-\tau)}\psi''(s,\tau)\,\mathrm{d}\tau + \frac{q}{EI}(\ell-s)\,\dot{\psi}(s,t) = 0,\end{aligned} \tag{S3.28a}$$

$$\begin{aligned}&\dot{\varphi}''(s,t) + \frac{\beta}{r\tau_m\tau_g}\int_{-\infty}^{t-\tau_r} e^{-\frac{1}{\tau_m}(t-\tau_r-\tau)}\varphi'(s,\tau)\,\mathrm{d}\tau\\ &\quad + \frac{\eta}{\bar{\tau}_m\tau_g}\int_{-\infty}^{t-\bar{\tau}_r} e^{-\frac{1}{\bar{\tau}_m}(t-\bar{\tau}_r-\tau)}\varphi''(s,\tau)\,\mathrm{d}\tau + \frac{q}{EI}(\ell-s)\,\dot{\varphi}(s,t) = 0,\end{aligned} \tag{S3.28b}$$

where equations (S3.27d) and (S3.27e) have been combined with the time derivatives of equations (S3.27a) and (S3.27c), respectively, while solving equation (S3.27b) with boundary conditions (S3.9b). We notice that equations (S3.28) along with the boundary conditions $(\text{S3.9a})_1$, $(\text{S3.9c})_1$ and (S3.12), form two equivalent decoupled problems. Moreover, up to a shift of $\pi/2$, such a problem is precisely the linearization of the planar model (S3.23) about the trivial solution $\theta \equiv 0$, which can be restated in dimensionless form as

$$\begin{aligned}&\dot{\hat{\theta}}''(\hat{s},\hat{t}) + \beta\frac{\ell}{r}\frac{\tau_s^2}{\tau_m\tau_g}\int_{-\infty}^{\hat{t}-\frac{\tau_r}{\tau_s}} e^{-\frac{\tau_s}{\tau_m}\left(\hat{t}-\frac{\tau_r}{\tau_s}-\tau\right)}\hat{\theta}'(\hat{s},\tau)\,\mathrm{d}\tau\\ &\quad + \eta\frac{\tau_s^2}{\bar{\tau}_m\tau_g}\int_{-\infty}^{\hat{t}-\frac{\bar{\tau}_r}{\tau_s}} e^{-\frac{\tau_s}{\bar{\tau}_m}\left(\hat{t}-\frac{\bar{\tau}_r}{\tau_s}-\tau\right)}\hat{\theta}''(\hat{s},\tau)\,\mathrm{d}\tau + \frac{q\ell^3}{K_1}(1-\hat{s})\dot{\hat{\theta}}(\hat{s},\hat{t}) = 0,\end{aligned} \tag{S3.29}$$

with boundary conditions

$$\hat{\theta}(0,\hat{t}) = 0, \tag{S3.30a}$$

$$\frac{\tau_g}{\tau_s^2}\dot{\hat{\theta}}'(1,\hat{t}) = -\frac{\beta}{\tau_m}\frac{\ell}{r}\int_{-\infty}^{\hat{t}-\frac{\tau_r}{\tau_s}} e^{-\frac{\tau_s}{\tau_m}\left(\hat{t}-\frac{\tau_r}{\tau_s}-\tau\right)}\hat{\theta}(1,\tau)\,\mathrm{d}\tau - \frac{\eta}{\bar{\tau}_m}\int_{-\infty}^{\hat{t}-\frac{\bar{\tau}_r}{\tau_s}} e^{-\frac{\tau_s}{\bar{\tau}_m}\left(\hat{t}-\frac{\bar{\tau}_r}{\tau_s}-\tau\right)}\hat{\theta}'(1,\tau)\,\mathrm{d}\tau, \tag{S3.30b}$$

for $\hat{t} > 0$, where $\hat{\theta}(\hat{s},\hat{t}) := \theta(\hat{s}\ell, \hat{t}\tau_s)$ for any fixed time scale τ_s. By seeking time-harmonic solutions of the form $\hat{\theta}(\hat{s},\hat{t}) = \Theta(\hat{s})e^{\hat{\omega}\hat{t}}$ with $\mathrm{Re}(\hat{\omega}) > -\min\{\tau_s/\tau_m, \tau_s/\bar{\tau}_m\}$, we get

$$a\Theta''(\hat{s}) + b\Theta'(\hat{s}) + c(1-\hat{s})\Theta(\hat{s}) = 0, \tag{S3.31}$$

where

$$a := \hat{\omega} + \eta\frac{\tau_s^2 e^{-\hat{\omega}\frac{\bar{\tau}_r}{\tau_s}}}{\tau_g\left(\bar{\tau}_m\hat{\omega}+\tau_s\right)}, \qquad b := \beta\frac{\ell}{r}\frac{\tau_s^2 e^{-\hat{\omega}\frac{\tau_r}{\tau_s}}}{\tau_g\left(\tau_m\hat{\omega}+\tau_s\right)}, \qquad \text{and} \qquad c := \hat{\omega}\frac{q\ell^3}{K_1}. \tag{S3.32}$$

By imposing the boundary conditions (S3.30), and neglecting the trivial case, we derive the following characteristic equation for the dimensionless frequency $\hat{\omega}$, namely,

$$\mathrm{Ai}(x_0)\left[b\,\mathrm{Bi}(x_1)+2\sqrt[3]{a^2c}\,\mathrm{Bi}'(x_1)\right]-\mathrm{Bi}(x_0)\left[b\,\mathrm{Ai}(x_1)+2\sqrt[3]{a^2c}\,\mathrm{Ai}'(x_1)\right]=0, \tag{S3.33}$$

where

$$x_0 := \frac{b^2-4ac}{4a\sqrt[3]{ac^2}}, \quad x_1 := \frac{b^2}{4a\sqrt[3]{ac^2}}, \tag{S3.34}$$

and $\mathrm{Ai}(x)$, $\mathrm{Bi}(x)$ are the Airy functions of the first and second kind, respectively, and a prime denotes differentiation of such functions with respect to their argument.

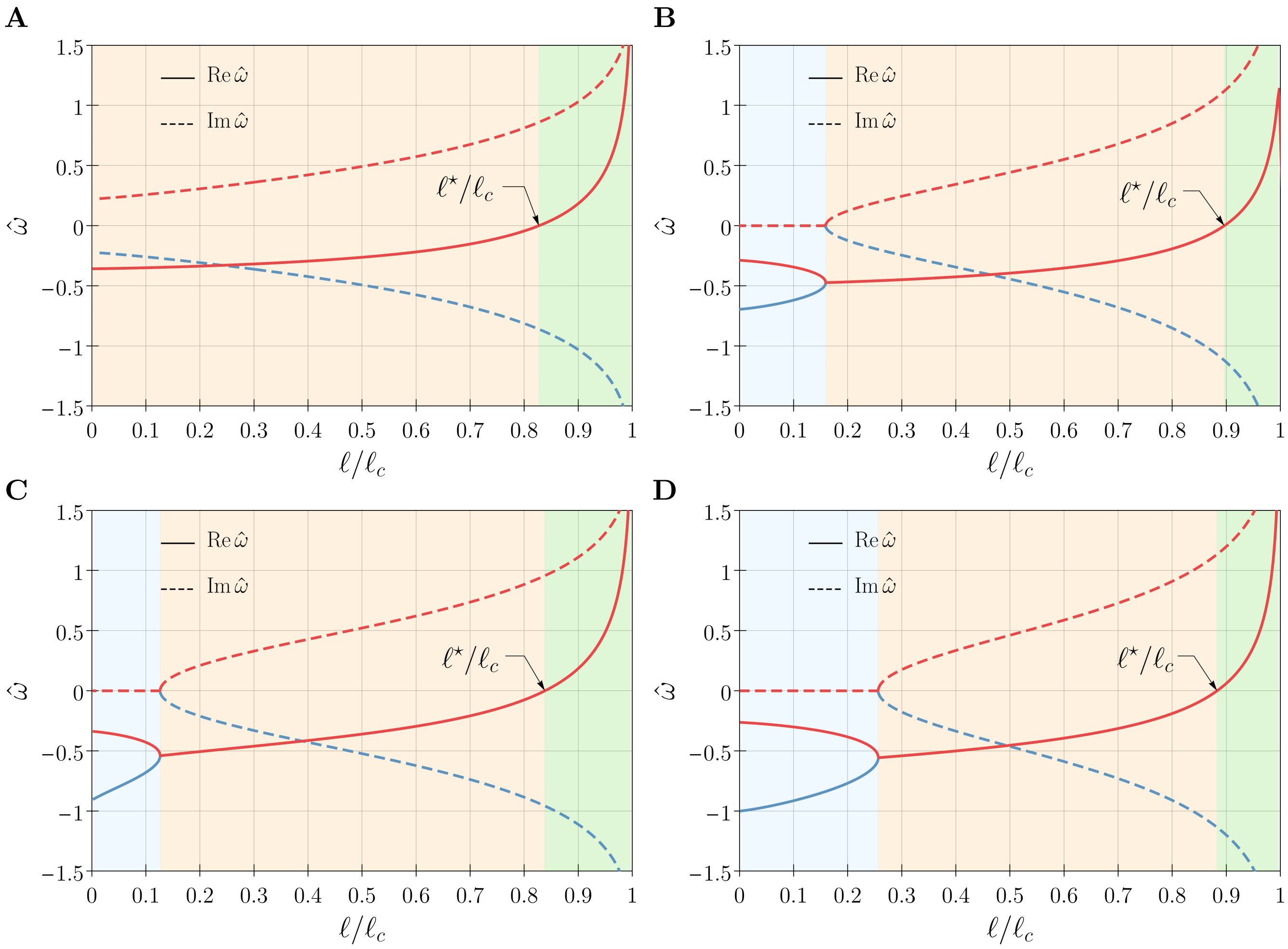

Figure S6. Real (solid lines) and imaginary (dashed lines) part of two roots of the characteristic equation (S3.33) as functions of $\ell/\ell_c \in [0,1]$ and for the model parameters introduced in the section. We distinguish three regions corresponding to different dynamical responses: (i) an exponential decay (light blue), (ii) a damped oscillation (orange), and (iii) an increasing oscillation (green) for $\ell > \ell^\star$. More specifically, we get **(A)** $\ell^\star \approx 0.827\,\ell_c$ for $\bar{\tau}_r = \bar{\tau}_m = 12$ min, **(B)** $\ell^\star \approx 0.896\,\ell_c$ for $\bar{\tau}_r = 1$ min and $\bar{\tau}_m = 12$ min, **(C)** $\ell^\star \approx 0.838\,\ell_c$ for $\bar{\tau}_r = 12$ min and $\bar{\tau}_m = 6$ min, and **(D)** $\ell^\star \approx 0.883\,\ell_c$ for $\bar{\tau}_r = 6$ min and $\bar{\tau}_m = 6$ min. For comparison, the same choice of model parameters yield a critical value of $\ell^\star \approx 0.895\,\ell_c$ in the gravitropic case (Agostinelli et al., 2020).

Then we can explore the stability of the model by performing a numerical study of the roots of equation (S3.33) for shoots of increasing length ℓ. To this aim, we exploited the FindRoot functionality of Mathematica v12.0.0.0. In agreement with the literature, we calibrated the model by setting $\beta = 0.8$, $\tau_g = 20\,\mathrm{h}$, $\tau_m = \tau_r = 12\,\mathrm{min}$, $r = 0.5\,\mathrm{mm}$, $E = 10^7$ Pa for the Young's modulus, and $\rho = 10^3\,\mathrm{Kg\,m^{-3}}$ for the mass density. As for η, $\bar{\tau}_r$ and $\bar{\tau}_m$, we estimated their order of magnitude by qualitatively fitting the steady-state solution (S3.26) and the dynamics reported by Okamoto et al. (2015), and we explored values around $\eta = 20$ and $\bar{\tau}_m = \bar{\tau}_r = 12\,\mathrm{min}$. For such a choice of model parameters, we determined the values of the frequency $\hat{\omega}$ letting ℓ range in $[0, \ell_c]$. Here, ℓ_c denotes the critical length at which an elastic rod of bending stiffness EI subject to a distributed vertical load of magnitude q loses stability, *i.e.*,

$$\ell_c := \sqrt[3]{\alpha_0 EI/q}, \tag{S3.35}$$

with $\alpha_0 \approx 7.837$ (Greenhill, 1881).

Fig. S6 shows the real (solid lines) and the imaginary (dashed lines) part of two roots of (S3.33). As for the case of the gravitropic rod model (Agostinelli et al., 2020), we distinguish in the figure three regions corresponding to different dynamical responses: (i) an exponential decay (light blue region, where roots are real and negative), (ii) a damped oscillation (orange region, where roots are complex conjugate with negative real part), and (iii) an increasing oscillation (green region, where roots are complex conjugate with positive real part) for $\ell > \ell^\star$. We remark the fact that the memory time $\bar{\tau}_m$ and the delay $\bar{\tau}_r$ influence the value of the critical length $\ell^\star$: Higher times $\bar{\tau}_r$ and $\bar{\tau}_m$ imply a lower critical length. This affects the overall effect of the proprioceptive term, which can either destabilize (Fig. S6A,C,D) or stabilize (Fig. S6B) the system with respect to the gravitropic case ($\eta = 0$).

S4 COMPUTATIONAL MODEL

We explored the nonlinear response of (S3.1) and (S3.5) by means of computational models implemented in Python3 codes available at https://github.com/mathLab/MorphoelasticRod, which are based on the FEniCS Project Version 2019.1.0 (Logg et al., 2012). In this section we describe only the numerical scheme for the full model (S3.1), since this can be easily adapted to its reduced version (S3.5).

An effective way to implement the model is to write all equations in the reference domain $\mathcal{B}_0$, *i.e.*, in terms of the parameter $S \in [0, \ell_0]$. Any material field can be converted into a spatial field, and vice versa. Indeed, as shown in Section S1.3, the motion $s(S,t)$ can be analytically determined for the growth law given by (S3.1b), namely,

$$s(S,t) = \begin{cases} S & \text{if } S \leq \ell_0 - \ell_g, \\ \ell(t^\star(S)) - \ell_g & \text{if } S > \ell_0 - \ell_g \text{ and } t \geq t^\star(S), \\ \ell(t) - (\ell_0 - S)e^{t/\tau_g} & \text{if } S > \ell_0 - \ell_g \text{ and } t < t^\star(S), \end{cases} \tag{S4.1}$$

where

$$\ell(t) = \begin{cases} \ell_0 e^{t/\tau_g} & \text{if } t \leq t^\star(0), \\ \max\{\ell_0, \ell_g\} + \frac{\ell_g}{\tau_g}\left(t - \max\{0, t^\star(0)\}\right) & \text{if } t > t^\star(0), \end{cases} \quad \text{and} \quad t^\star(S) = \tau_g \ln\left(\frac{\ell_g}{\ell_0 - S}\right). \tag{S4.2}$$

Moreover, its inverse is given by

$$S(s,t) := \begin{cases} s & \text{if } s \leq \ell_0 - \ell_g, \\ \ell_0 + \left[s - \ell\left(\bar{t}^\star(s,t)\right)\right] e^{-\bar{t}^\star(s,t)/\tau_g} & \text{if } s \in \left(\ell_0 - \ell_g, \ell(t) - \ell_g\right], \\ \ell_0 + \left[s - \ell\left(t\right)\right] e^{-t/\tau_g} & \text{if } s \in \left(\ell(t) - \ell_g, \ell(t)\right], \end{cases} \tag{S4.3}$$

where $\bar{t}^\star(s,t) := t + \tau_g\left(s + \ell_g - \ell(t)\right)/\ell_g$.

As a first step towards the numerical formulation, we introduce some auxiliary fields representing the delay integrals, namely,

$$w_{1,g}(S,t) := -\frac{1}{\tau_m}\int_{-\infty}^{t-\tau_r} e^{-\frac{1}{\tau_m}(t-\tau_r-\tau)} \sin\theta_h(S,\tau)\cos\alpha_h(S,\tau)\,\mathrm{d}\tau, \tag{S4.4a}$$

$$w_{1,p}(S,t) := -\frac{1}{\bar{\tau}_m}\int_{-\infty}^{t-\bar{\tau}_r} e^{-\frac{1}{\bar{\tau}_m}(t-\bar{\tau}_r-\tau)} u_1(S,\tau)\,\mathrm{d}\tau, \tag{S4.4b}$$

$$w_{2,g}(S,t) := \frac{1}{\tau_m}\int_{-\infty}^{t-\tau_r} e^{-\frac{1}{\tau_m}(t-\tau_r-\tau)} \cos\theta_h(S,\tau)\,\mathrm{d}\tau, \tag{S4.4c}$$

$$w_{2,p}(S,t) := -\frac{1}{\bar{\tau}_m}\int_{-\infty}^{t-\bar{\tau}_r} e^{-\frac{1}{\bar{\tau}_m}(t-\bar{\tau}_r-\tau)} u_2(S,\tau)\,\mathrm{d}\tau, \tag{S4.4d}$$

so that such integrals may be computed from the solution of the following differential equations

$$\frac{\mathrm{d}w_{1,g}}{\mathrm{d}t} = -\frac{1}{\tau_m}w_{1,g} - \frac{1}{\tau_m}\sin\theta_h(S,t-\tau_r)\cos\alpha_h(S,t-\tau_r), \tag{S4.5a}$$

$$\frac{\mathrm{d}w_{1,p}}{\mathrm{d}t} = -\frac{1}{\bar{\tau}_m}w_{1,p} - \frac{1}{\bar{\tau}_m}u_1(S,t-\bar{\tau}_r), \tag{S4.5b}$$

$$\frac{\mathrm{d}w_{2,g}}{\mathrm{d}t} = -\frac{1}{\tau_m} w_{2,g} + \frac{1}{\tau_m}\cos\theta_h(S, t-\tau_r), \tag{S4.5c}$$

$$\frac{\mathrm{d}w_{2,p}}{\mathrm{d}t} = -\frac{1}{\bar{\tau}_m} w_{2,p} - \frac{1}{\bar{\tau}_m} u_2(S, t-\bar{\tau}_r), \tag{S4.5d}$$

respectively. Then we can write the governing equations in terms of the Euler angles introduced in S3.2 and the angles describing the statoliths pile configuration as defined in S2, *i.e.*,

$$\frac{\partial m_1}{\partial S} = -\, q\,[\ell(t) - s(S,t)]\,\lambda\cos\psi, \tag{S4.6a}$$

$$\frac{\partial m_2}{\partial S} = 0, \tag{S4.6b}$$

$$\frac{\partial m_3}{\partial S} = q\,[\ell(t) - s(S,t)]\,\lambda\sin\psi\cos\varphi, \tag{S4.6c}$$

$$\begin{aligned}\tau_a\frac{\mathrm{d}\theta_h}{\mathrm{d}t} =& \cos\theta_h\,[\cos\chi\cos\alpha_h\cos\varphi + (-\cos\psi\cos\alpha_h\sin\chi + \sin\psi\sin\alpha_h)\sin\varphi] \\ &- (\cos\varphi\sin\chi + \cos\chi\cos\psi\sin\varphi)\sin\theta_h,\end{aligned} \tag{S4.6d}$$

$$\tau_a\frac{\mathrm{d}\alpha_h}{\mathrm{d}t}\sin\theta_h = -\cos\chi\cos\varphi\sin\alpha_h + (\cos\alpha_h\sin\psi + \cos\psi\sin\chi\sin\alpha_h)\sin\varphi, \tag{S4.6e}$$

$$\frac{\mathrm{d}u_1^\star}{\mathrm{d}t} = \frac{1}{\lambda}\frac{\mathrm{d}\lambda}{\mathrm{d}t}\left[\frac{\alpha}{r}\cos\left(\frac{2\pi t}{\tau_e}\right) + \frac{\beta}{r} w_{1,g} + \eta w_{1,p}\right], \tag{S4.6f}$$

$$\frac{\mathrm{d}u_2^\star}{\mathrm{d}t} = \frac{1}{\lambda}\frac{\mathrm{d}\lambda}{\mathrm{d}t}\left[\frac{\alpha}{r}\sin\left(\frac{2\pi t}{\tau_e}\right) + \frac{\beta}{r} w_{2,g} + \eta w_{2,p}\right], \tag{S4.6g}$$

where $\lambda(S,t) = \frac{\partial s(S,t)}{\partial S}$ and

$$\begin{aligned} m_1 =& EI\,\{\cos\psi\cos\varphi\,[(u_1 - u_1^\star)\cos\chi - (u_2 - u_2^\star)\sin\chi] \\ &- \sin\varphi\,[(u_1 - u_1^\star)\sin\chi + (u_2 - u_2^\star)\cos\chi]\} + \mu J\,(u_3 - u_3^\star)\sin\psi\cos\varphi, \end{aligned} \tag{S4.7a}$$

$$\begin{aligned} m_2 =& EI\,\{\cos\psi\sin\varphi\,[(u_1 - u_1^\star)\cos\chi - (u_2 - u_2^\star)\sin\chi] \\ &+ \cos\varphi\,[(u_1 - u_1^\star)\sin\chi + (u_2 - u_2^\star)\cos\chi]\} + \mu J\,(u_3 - u_3^\star)\sin\psi\sin\varphi, \end{aligned} \tag{S4.7b}$$

$$m_3 = -\,EI\sin\psi\,[(u_1 - u_1^\star)\cos\chi - (u_2 - u_2^\star)\sin\chi] + \mu J\,(u_3 - u_3^\star)\cos\psi, \tag{S4.7c}$$

with

$$u_1 = \frac{1}{\lambda}\left[\frac{\partial\psi}{\partial S}\sin\chi - \frac{\partial\varphi}{\partial S}\cos\chi\sin\psi\right], \tag{S4.8a}$$

$$u_2 = \frac{1}{\lambda}\left[\frac{\partial\psi}{\partial S}\cos\chi + \frac{\partial\varphi}{\partial S}\sin\chi\sin\psi\right], \tag{S4.8b}$$

$$u_3 = \frac{1}{\lambda}\left[\frac{\partial\chi}{\partial S} + \frac{\partial\varphi}{\partial S}\cos\psi\right]. \tag{S4.8c}$$

The weak formulation of (S4.5)-(S4.6) is obtained by multiplying such equations by the test functions and integrating by parts in space along the interval $[0, \ell_0]$ while accounting for the appropriate boundary conditions. Following standard finite element procedures, the unknowns are discretized in space using

linear Langrange shape functions, while for the time discretization we used the backward Euler method. Finally, the rod axis $\mathbf{p}$ can be reconstructed by integrating in space the tangent that, for unshearable rods, coincides with the director $\mathbf{d}_3$, *i.e.*,

$$\mathbf{p}(S,t) = \mathbf{p}(0,t) + \int_0^S \lambda(\zeta,t)\mathbf{d}_3(\zeta,t)\,\mathrm{d}\zeta. \tag{S4.9}$$

S5 EXPERIMENTAL MATERIALS AND METHODS

Observations of three specimens of *Arabidopsis thaliana* (ecotype Col-0, stock N1093 from NASC) were conducted as a proof of principle, *i.e.*, as a qualitative check of the plausibility of our theoretical findings.

The experiments were carried out in a growth and observation chamber of size $43 \times 43 \times 40$ cm, which does not allow to actively control temperature and humidity. However, the chamber was placed in a controlled environment and we regularly monitored temperature (23-25°C) and relative humidity (45-60%).

The specimens of *Arabidopsis thaliana* were grown (individually) from seeds in square pots with perforated bottom of size $7 \times 7 \times 8$ cm. As for the growing medium, we used the peat based soil amendment Optimus Universal from Sementi Dotto SDD S.p.a., which was mixed with lightweight expanded clay aggregate. Plants were watered regularly with a solution of tap water containing a commercial fertilizer (Vanity NPK 6-5-9 from Bayer Garden, $\sim 0.1\%$ v/v).

Plants were grown under continuous light provided by a LED light panel (from Esbaybulbs, model bazw0173cn) of size 31×31 cm installed at the top of the observation chamber. This resulted in a light intensity of about $7\,\mathrm{W/m^2}$ as measured at the center of the observation chamber with a spectrometer (from Ocean Insight, model flms05233).

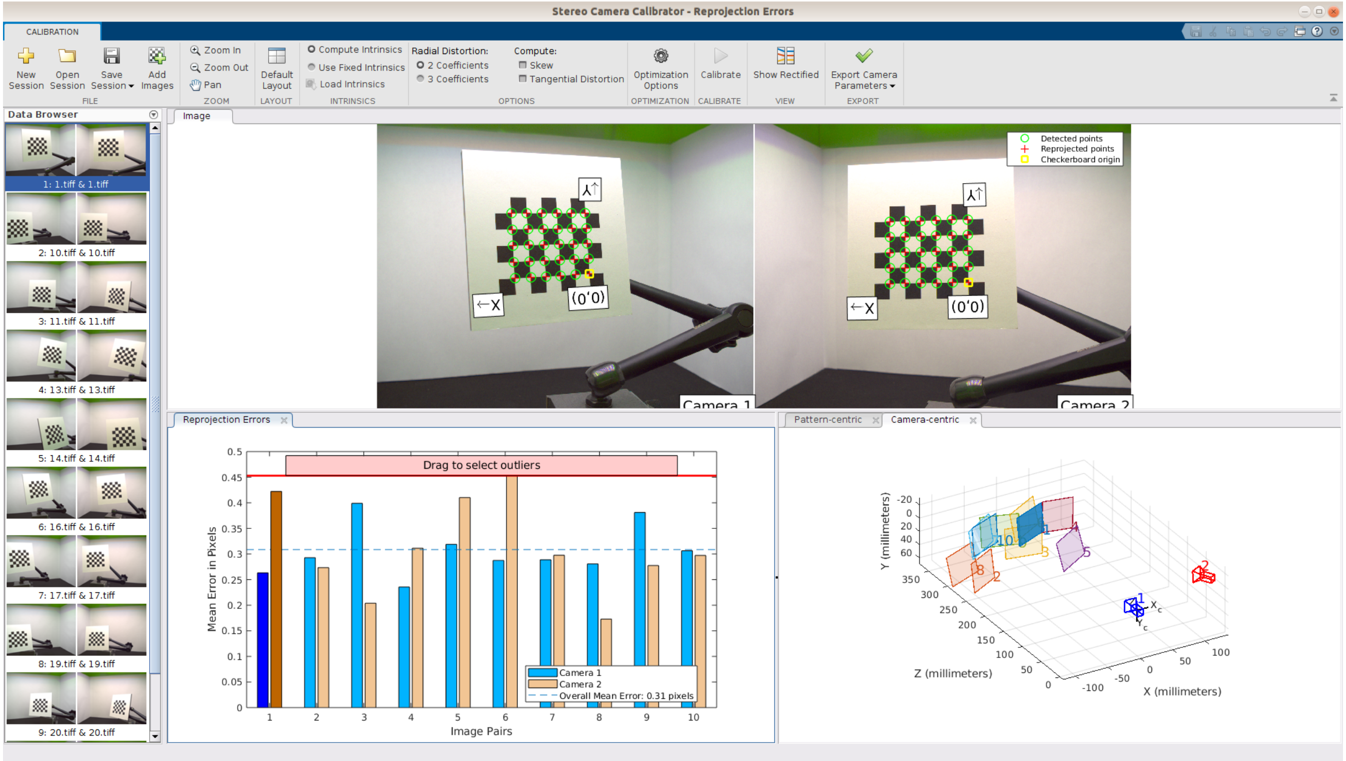

Figure S7. Results of the stereo imaging system calibration, as produced by the MATLAB Stereo Camera Calibrator App.

As for data collection and processing, images were acquired by using two digital cameras, namely, two acA4024-29uc c mount cameras from Basler, both of which were equipped with an objective m0824-mpw2 from Computar. We calibrated the stereo imaging system by exploiting the Computer Vision Toolbox in MATLAB R2019b. After setting the two cameras, we acquired 20 image pairs of a 6×7 calibration checkerboard in different positions in the scene. By means of the MATLAB Stereo Camera Calibrator App,

we performed the calibration with 10 pictures that were selected to minimize the overall mean reprojection error (0.3086 pixels), see Fig. S7. In view of the qualitative scope of our experiments, we opted for a manual tracking of the plant tip on the stereo pair of images, which were acquired at a rate of 1 frame per minute. Finally, we exploited the triangulate function of MATLAB R2019b to compute the 3D locations corresponding to the selected matching pairs of points.

REFERENCES

Agostinelli, D., Lucantonio, A., Noselli, G., and DeSimone, A. (2020). Nutations in growing plant shoots: The role of elastic deformations due to gravity loading. *Journal of the Mechanics and Physics of Solids* 136, 103702. doi:10.1016/j.jmps.2019.103702. The Davide Bigoni 60th Anniversary Issue

Antman, S. (2005). *Nonlinear Problems of Elasticity* (Springer-Verlag New York). doi:10.1007/0-387-27649-1

Bastien, R., Douady, S., and Moulia, B. (2014). A unifying modeling of plant shoot gravitropism with an explicit account of the effects of growth. *Frontiers in plant science* 5, 136

Bastien, R. and Meroz, Y. (2016). The kinematics of plant nutation reveals a simple relation between curvature and the orientation of differential growth. *PLoS computational biology* 12, e1005238. doi:10.1371/journal.pcbi.1005238

Chauvet, H., Moulia, B., Legué, V., Forterre, Y., and Pouliquen, O. (2019). Revealing the hierarchy of processes and time-scales that control the tropic response of shoots to gravi-stimulations. *Journal of Experimental Botany* 70, 1955–1967

Chelakkot, R. and Mahadevan, L. (2017). On the growth and form of shoots. *Journal of The Royal Society Interface* 14, 20170001

Erickson, R. and Sax, K. (1956). Elemental growth rate of the primary root of zea mays. *Proceedings of the American Philosophical Society* 100, 487–498

Greenhill, A. (1881). Determination of the greatest height consistent with stability that a vertical pole or mast can be made, and of the greatest height to which a tree of given proportions can grow. *Proceedings of the Cambridge Philosophical Society* 4, 65–73

Hale, J. and Lunel, S. (1993). *Introduction to functional differential equations* (Springer Science & Business Media)

Logg, A., Mardal, K.-A., and Wells, G. (2012). *Automated solution of differential equations by the finite element method: The FEniCS book*, vol. 84 (Berlin, Heidelberg: Springer Berlin Heidelberg). doi:10.1007/978-3-642-23099-8

Meroz, Y., Bastien, R., and Mahadevan, L. (2019). Spatio-temporal integration in plant tropisms. *Journal of the Royal Society Interface* 16, 20190038

Nakamura, M., Nishimura, T., and Morita, M. (2019). Gravity sensing and signal conversion in plant gravitropism. *Journal of experimental botany* 70, 3495–3506. doi:10.1093/jxb/erz158

Okamoto, K., Ueda, H., Shimada, T., Tamura, K., Kato, T., Tasaka, M., et al. (2015). Regulation of organ straightening and plant posture by an actin–myosin XI cytoskeleton. *Nature Plants* 1, 15031. doi:10.1038/nplants.2015.31

Paul-Victor, C. and Rowe, N. (2011). Effect of mechanical perturbation on the biomechanics, primary growth and secondary tissue development of inflorescence stems of arabidopsis thaliana. *Annals of botany* 107, 209–218. doi:10.1093/aob/mcq227

Porat, A., Tedone, F., Palladino, M., Marcati, P., and Meroz, Y. (2020). A general 3d model for growth dynamics of sensory-growth systems: From plants to robotics. *Frontiers in Robotics and AI* 7, 89. doi:10.3389/frobt.2020.00089

Somolinos, A. (1978). Periodic solutions of the sunflower equation: $\ddot{x} + (a/r)x + (b/r)\sin x(t - r) = 0$. *Quarterly of Applied Mathematics* 35, 465–478. doi:doi.org/10.1090/qam/465265

von Sachs, J. (1882). Über orthotrope und plagiotrope Pflanzentheile. *Arbeiten des Botanischen Instituts in Würzburg* 2, 226–284
